# Supplementary material for: Decoding individual identity from brain activity elicited in imagining common experiences
Source: Nat Commun. 2020 Nov 20;11:5916. doi: 10.1038/s41467-020-19630-y (PMC7679397; doi:10.1038/s41467-020-19630-y)
Supplement: Supplementary file 1 — Supplementary Information [file 41467_2020_19630_MOESM1_ESM.pdf]

Supplementary Materials

Contents **Page**

**Supplementary Figure 1.** Mean±SD ratings of the likelihood of each scenario to have occurred and the vividness of the mental image formed (26 participants).	4
**Supplementary Table 1.** Complete listing of RSA results for all ROIs, serving as a companion to **Figures 3** and **4**.	5
**Supplementary Table 2.** Complete listing of ‘individual-differences” analyses for all ROIs, serving as a companion to **Figure 3 and 6**.	7
**Supplementary Figure 2**. Both the verbal and attribute models contributed to predicting person-specific fMRI representations.	9
**Supplementary Figure 3.** Replication of **Figure 3** “fMRI activation patterns elicited in imagining common scenarios reflect person-specific information” and **Figure 4** when differently analyses were computed using the Verbal models only.	10
**Supplementary Figure 4.** Replication of **Figure 3** “fMRI activation patterns elicited in imagining common scenarios reflect person-specific information” and **Figure 4** when differently analyses were computed using the Attribute models only.	11
**Supplementary Figure 5**. Replication of **Figure 3** “fMRI activation patterns elicited in imagining common scenarios reflect person-specific information” and **Figure 4** when differently group-average model representations were computed by taking the pointwise mean of personal models in model feature space.	12
**Supplementary Figure 6.** Replication of **Figure 3** “fMRI activation patterns elicited in imagining common scenarios reflect person-specific information” and **Figure 4** when differently the analyses were performed on 50 rather than 100 voxels per ROI.	13
**Supplementary Figure 7.** Replication of **Figure 3** “fMRI activation patterns elicited in imagining common scenarios reflect person-specific information” and **Figure 4** when differently the analyses were performed on 200 rather than 100 voxels per ROI.	14
**Supplementary Figure 8.** Comparative RSA results to **Figure 3** “fMRI activation patterns elicited in imagining common scenarios reflect person-specific information” when participants’ fMRI data was compared to a generic distributional semantic model representations (GloVe) of the words in the stimulus prompt.	15
**Supplementary Figure 9.** Replication of **Figure 3** “fMRI activation patterns elicited in imagining common scenarios reflect person-specific information” and **Figure 4** when differently ROIs in left and right brain hemispheres of the five left handed participants brains were swapped prior to analyses to counteract possible effects surrounding lateralized language function.	16

Supplementary Table 3. Companion data to **Figure 5** “Neuroanatomical distribution of person-specific representational structure (RSA-Searchlight)”. Listing of significant clusters for Searchlight Partial RSA: fMRI vs Personal Models controlling for Group models. 17

Supplementary Table 4. Companion data to **Figure 5** “Neuroanatomical distribution of person-specific representational structure (RSA-Searchlight)”. Listing of significant clusters for Searchlight RSA: fMRI vs Personal Models. 18

Supplementary Table 5. Companion data to **Figure 5** “Neuroanatomical distribution of person-specific representational structure (RSA-Searchlight)”. Listing of significant clusters for Searchlight Partial RSA: fMRI vs Group Models. 21

Supplementary Figure 10. Replication of **Figure 6** “Individual identity can be decoded from fMRI activity elicited during the imagination of common scenarios” when the analysis was performed using verbal and attribute models in isolation. 22

Supplementary Figure 11. Replication of **Figure 6** “Individual identity can be decoded from fMRI activity elicited in imagining personal experiences” when *differently* the analysis was performed on 50 rather than 100 voxels per ROI. 23

Supplementary Figure 12. Replication of **Figure 6** “Individual identity can be decoded from fMRI activity elicited in imagining personal experiences” when *differently* the analysis was performed on 200 rather than 100 voxels per ROI. 24

Supplementary Figure 13. Replication of **Figure 6** “Individual identity can be decoded from fMRI activity elicited in imagining personal experiences” when differently the analysis was performed on the subset of 18 participants with “high fMRI signal”. 25

Supplementary Figure 14. Replication of **Figure 6** “Individual identity can be decoded from fMRI activity elicited in imagining personal experiences” when *differently* the analysis was performed on the subset of 17 females. 26

Supplementary Figure 15. Replication of **Figure 6** “Individual identity can be decoded from fMRI activity elicited in imagining personal experiences” when *differently* the analysis was performed on the subset of 9 males. 27

Supplementary Figure 16. Replication of **Supplementary Figure 2** “Both the verbal and attribute models contributed to explaining fMRI activity in each ROI” when differently analyses were performed using 50 rather than 100 voxels per ROI. 28

Supplementary Figure 17. Replication of **Supplementary Figure 2** “Both the verbal and attribute models contributed to explaining fMRI activity in each ROI” when differently analyses were performed using 200 rather than 100 voxels per ROI. 29

Supplementary Figure 18. Estimated head translation and rotation across the duration of the experiment. 30

Supplementary Figure 19. Different participants were characterized by different hemodynamic response functions (HRF). 31

Supplementary Figure 20. Different participants were characterized by different hemodynamic response functions (HRF). The plot replicates the top row (spike) of **Supplementary Figure 19**, this time showing data for all 26 participants. 32

Supplementary Figure 21. Different participants were characterized by different hemodynamic response functions (HRF). The plot replicates the top row (boxcar) of **Supplementary Figure 19**, this time showing data for all 26 participants. 33

Supplementary Table 6. Detailed protocol for rating experiential attributes and scenario vividness and likelihood. 34

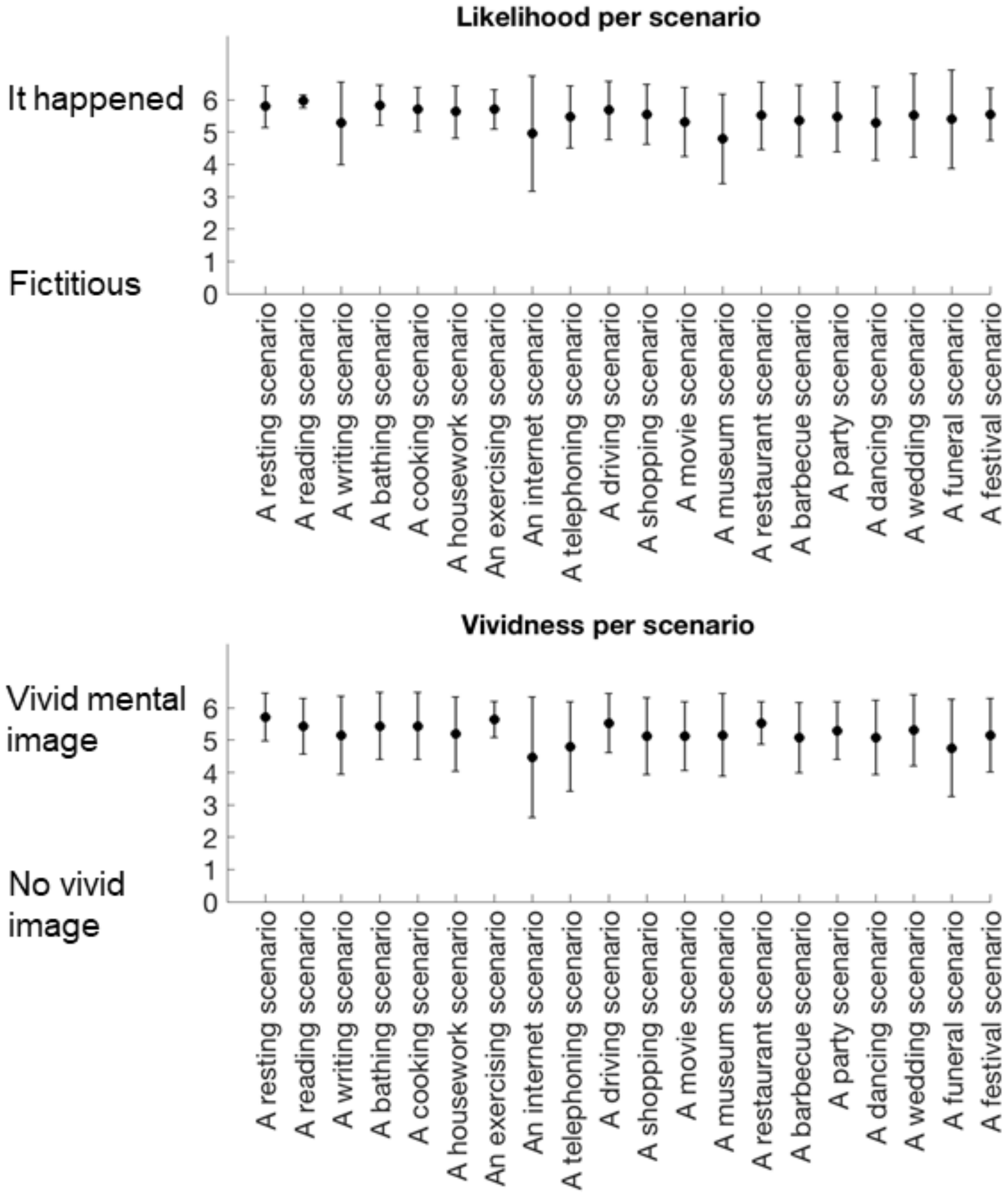

Supplementary Figure 1. Mean±SD ratings of the likelihood of each scenario to have occurred and the vividness of the mental image formed (26 participants): Because we could not guarantee in advance that participants would have first-hand experience of the different scenarios, we estimated this by having participants rate each imaginary scenario on a Likert scale of 0 (fictitious) to 6 (it has happened). We also asked them to rate how vividly each scenario was imagined (0-6, 6=vivid). See “Detailed protocol for rating experiential attributes and scenario vividness and likelihood” in **Supplementary Table 6** for details of the rating protocol.

Supplementary Table 1. Complete listing of RSA results for all ROIs, serving as a companion to **Figure 4**. The leftmost (second to seventh) columns cover RSA results comparing each individual's fMRI data to their own personal multimodal model. The rightmost columns (ninth to fourteenth) cover RSA results comparing each individual's fMRI data to a group-average multimodal model built from other participants (e.g. participant 1's fMRI would be compared to a group-model corresponding to participants 2 to 26). t>0 and p correspond to the t-statistic and uncorrected p-value associated with one sample t-tests against zero (1-tailed). fdrP corresponds to False Discovery Rate (FDR) corrected p-values across all ROIs. d corresponds to Cohen's d, which was computed by dividing the t-statistic by $26^{1/2}$. Rows shaded in grey correspond to the 8 ROIs that in the fMRI vs group model test returned FDR corrected p-values less than p=0.05 (across all 90 ROIs, see also **Figures 3 and 4**).

ROI	avg(rOwnMdl)	SD(rOwnMdl)	t>0	p	fdrP	d		avg(rGrpMdl)	SD(rGrpMdl)	t>0	p	fdrP	d
Precuneus_L	0.1450	0.1375	5.3779	0.0000	0.0024	1.0547		0.1446	0.1476	4.9963	0.0000	0.0070	0.9799
Precuneus_R	0.1265	0.1373	4.6981	0.0000	0.0047	0.9214		0.1067	0.1167	4.6627	0.0000	0.0070	0.9144
Temporal_Mid_L	0.1183	0.1277	4.7233	0.0000	0.0047	0.9263		0.1142	0.1335	4.3619	0.0001	0.0111	0.8554
Parietal_Inf_L	0.1041	0.1268	4.1882	0.0002	0.0116	0.8214		0.1006	0.1226	4.1857	0.0002	0.0140	0.8209
Cingulum_Post_L	0.1027	0.1004	5.2165	0.0000	0.0024	1.0230		0.0994	0.1089	4.6517	0.0000	0.0070	0.9123
Temporal_Mid_R	0.0973	0.1406	3.5297	0.0008	0.0327	0.6922		0.0764	0.1350	2.8862	0.0040	0.1510	0.5660
Occipital_Mid_L	0.0952	0.1182	4.1046	0.0002	0.0124	0.8050		0.0947	0.1199	4.0290	0.0002	0.0150	0.7901
Frontal_Mid_L	0.0933	0.1186	4.0110	0.0002	0.0138	0.7866		0.0744	0.1054	3.5993	0.0007	0.0393	0.7059
Angular_L	0.0911	0.1302	3.5696	0.0007	0.0327	0.7000		0.0974	0.1220	4.0724	0.0002	0.0150	0.7987
Frontal_Sup_L	0.0807	0.1101	3.7385	0.0005	0.0246	0.7332		0.0626	0.1263	2.5264	0.0091	0.2984	0.4955
Angular_R	0.0787	0.1306	3.0727	0.0025	0.0610	0.6026		0.0708	0.1194	3.0233	0.0029	0.1306	0.5929
Frontal_Med_Orb_L	0.0674	0.0757	4.5355	0.0001	0.0057	0.8895		0.0482	0.0848	2.8976	0.0039	0.1510	0.5683
Frontal_Sup_Medial_L	0.0672	0.1044	3.2798	0.0015	0.0437	0.6432		0.0548	0.1106	2.5255	0.0091	0.2984	0.4953
Cingulum_Post_R	0.0645	0.0956	3.4394	0.0010	0.0354	0.6745		0.0531	0.0864	3.1366	0.0022	0.1102	0.6151
Frontal_Inf_Orb_L	0.0605	0.0879	3.5120	0.0009	0.0327	0.6888		0.0281	0.0820	1.7489	0.0463	1.1144	0.3430
Frontal_Sup_Medial_R	0.0600	0.1052	2.9067	0.0038	0.0863	0.5701		0.0330	0.1155	1.4568	0.0788	1.3581	0.2857
Parietal_Sup_L	0.0560	0.1080	2.6426	0.0070	0.1524	0.5182		0.0460	0.0995	2.3544	0.0134	0.3819	0.4617
Frontal_Inf_Tri_L	0.0559	0.0897	3.1799	0.0020	0.0496	0.6236		0.0472	0.0967	2.4856	0.0100	0.3046	0.4875
Cuneus_L	0.0556	0.0854	3.3197	0.0014	0.0422	0.6510		0.0176	0.0880	1.0202	0.1587	1.9619	0.2001
Frontal_Mid_R	0.0525	0.0830	3.2282	0.0017	0.0467	0.6331		0.0177	0.1010	0.8932	0.1901	2.1742	0.1752
Precentral_L	0.0525	0.0783	3.4176	0.0011	0.0354	0.6703		0.0223	0.0703	1.6191	0.0590	1.2264	0.3175
SupraMarginal_L	0.0525	0.1237	2.1624	0.0202	0.2670	0.4241		0.0465	0.1047	2.2661	0.0162	0.4353	0.4444
Frontal_Sup_R	0.0475	0.1122	2.1566	0.0204	0.2670	0.4229		0.0324	0.1001	1.6489	0.0558	1.2163	0.3234
Temporal_Inf_L	0.0469	0.1107	2.1620	0.0202	0.2670	0.4240		0.0289	0.1108	1.3294	0.0979	1.4505	0.2607
Occipital_Mid_R	0.0449	0.0959	2.3890	0.0124	0.2360	0.4685		0.0223	0.0866	1.3136	0.1004	1.4505	0.2576
Cingulum_Mid_L	0.0406	0.0866	2.3928	0.0123	0.2360	0.4693		0.0409	0.1037	2.0101	0.0277	0.7030	0.3942
Parietal_Sup_R	0.0395	0.0833	2.4184	0.0116	0.2360	0.4743		0.0223	0.0725	1.5712	0.0644	1.2799	0.3081
Cingulum_Ant_L	0.0394	0.0898	2.2370	0.0172	0.2670	0.4387		0.0082	0.0999	0.4160	0.3405	2.7705	0.0816
Rectus_L	0.0392	0.0914	2.1833	0.0193	0.2670	0.4282		0.0221	0.0744	1.5133	0.0714	1.3059	0.2968
Frontal_Inf_Orb_R	0.0375	0.0867	2.2039	0.0185	0.2670	0.4322		0.0164	0.0702	1.1913	0.1224	1.6461	0.2336
Supp_Motor_Area_L	0.0368	0.0853	2.1990	0.0187	0.2670	0.4313		0.0058	0.0780	0.3772	0.3546	2.7705	0.0740
ParaHippocampal_L	0.0360	0.0775	2.3673	0.0130	0.2376	0.4643		0.0105	0.0862	0.6187	0.2709	2.5049	0.1213
Frontal_Mid_Orb_L	0.0351	0.0809	2.2084	0.0183	0.2670	0.4331		0.0194	0.0715	1.3831	0.0894	1.4465	0.2712
Calcarine_L	0.0349	0.0797	2.2341	0.0173	0.2670	0.4381		0.0223	0.0741	1.5320	0.0690	1.3059	0.3005
Hippocampus_R	0.0316	0.0868	1.8584	0.0375	0.4510	0.3645		0.0106	0.0815	0.6633	0.2566	2.5049	0.1301
Hippocampus_L	0.0307	0.0691	2.2652	0.0162	0.2670	0.4442		0.0059	0.0815	0.3697	0.3573	2.7705	0.0725
Frontal_Mid_Orb_R	0.0302	0.0796	1.9322	0.0324	0.4113	0.3789		-0.0011	0.0683	-0.0794	0.5313	3.5741	-0.0156
Temporal_Pole_Mid_L	0.0292	0.0791	1.8852	0.0355	0.4394	0.3697		0.0161	0.0862	0.9534	0.1747	2.1036	0.1870
Postcentral_L	0.0289	0.0848	1.7356	0.0475	0.5297	0.3404		0.0145	0.0910	0.8106	0.2126	2.3723	0.1590
Frontal_Inf_Oper_L	0.0277	0.0835	1.6909	0.0516	0.5494	0.3316		0.0231	0.1049	1.1209	0.1365	1.7839	0.2198
Supp_Motor_Area_R	0.0272	0.0958	1.4463	0.0803	0.7648	0.2836		0.0156	0.0610	1.3075	0.1015	1.4505	0.2564
Calcarine_R	0.0271	0.0966	1.4304	0.0825	0.7701	0.2805		0.0088	0.0947	0.4728	0.3202	2.7638	0.0927
Precentral_R	0.0270	0.0815	1.6924	0.0515	0.5494	0.3319		0.0024	0.0764	0.1594	0.4373	3.2111	0.0313
ParaHippocampal_R	0.0263	0.0820	1.6386	0.0569	0.5872	0.3214		0.0008	0.0873	0.0476	0.4812	3.4394	0.0093
Occipital_Sup_R	0.0256	0.0737	1.7739	0.0441	0.5176	0.3479		0.0137	0.0685	1.0225	0.1582	1.9619	0.2005
Fusiform_R	0.0256	0.0815	1.6019	0.0609	0.5924	0.3142		0.0081	0.0677	0.6096	0.2738	2.5049	0.1196

Parietal_Inf_R	0.0251	0.0736	1.7430	0.0468	0.5297	0.3418		0.0230	0.0706	1.6631	0.0544	1.2163	0.3262
Temporal_Inf_R	0.0251	0.0929	1.3751	0.0906	0.8130	0.2697		-0.0097	0.0729	-0.6803	0.7487	4.3352	-0.1334
Temporal_Sup_R	0.0244	0.0921	1.3520	0.0942	0.8157	0.2652		0.0234	0.0823	1.4470	0.0802	1.3581	0.2838
Cingulum_Mid_R	0.0236	0.0917	1.3118	0.1008	0.8379	0.2573		0.0069	0.0794	0.4432	0.3307	2.7705	0.0869
SupraMarginal_R	0.0233	0.1170	1.0138	0.1602	1.1274	0.1988		0.0284	0.1058	1.3682	0.0917	1.4465	0.2683
Occipital_Sup_L	0.0227	0.0715	1.6185	0.0590	0.5872	0.3174		-0.0021	0.0597	-0.1836	0.5721	3.6534	-0.0360
Thalamus_L	0.0222	0.0822	1.3794	0.0900	0.8130	0.2705		0.0028	0.0524	0.2705	0.3945	2.9583	0.0531
Frontal_Med_Orb_R	0.0217	0.0953	1.1599	0.1285	0.9798	0.2275		0.0184	0.1014	0.9261	0.1816	2.1302	0.1816
Rolandic_Oper_L	0.0212	0.0665	1.6290	0.0579	0.5872	0.3195		0.0099	0.0679	0.7446	0.2317	2.4274	0.1460
Rectus_R	0.0199	0.0756	1.3457	0.0952	0.8157	0.2639		0.0045	0.0421	0.5486	0.2941	2.5870	0.1076
Cingulum_Ant_R	0.0199	0.0756	1.3391	0.0963	0.8157	0.2626		0.0127	0.0862	0.7535	0.2291	2.4274	0.1478
Pallidum_L	0.0180	0.0849	1.0814	0.1449	1.0691	0.2121		-0.0233	0.0742	-1.6037	0.9393	4.9388	-0.3145
Frontal_Inf_Tri_R	0.0171	0.0733	1.1863	0.1233	0.9561	0.2327		-0.0033	0.0883	-0.1912	0.5750	3.6534	-0.0375
Postcentral_R	0.0169	0.0899	0.9595	0.1733	1.1876	0.1882		0.0121	0.0836	0.7387	0.2335	2.4274	0.1449
Fusiform_L	0.0165	0.0647	1.3003	0.1027	0.8387	0.2550		-0.0036	0.0725	-0.2567	0.6002	3.7168	-0.0503
Frontal_Sup_Orb_R	0.0158	0.0641	1.2533	0.1108	0.8896	0.2458		-0.0017	0.0604	-0.1452	0.5571	3.6534	-0.0285
Insula_L	0.0150	0.0728	1.0518	0.1515	1.0826	0.2063		0.0062	0.0764	0.4129	0.3416	2.7705	0.0810
Cuneus_R	0.0146	0.0675	1.1047	0.1399	1.0492	0.2166		0.0082	0.0657	0.6349	0.2656	2.5049	0.1245
Frontal_Inf_Oper_R	0.0146	0.0600	1.2367	0.1138	0.8978	0.2425		-0.0007	0.0616	-0.0550	0.5217	3.5741	-0.0108
Paracentral_Lobule_L	0.0142	0.0755	0.9566	0.1740	1.1876	0.1876		-0.0031	0.0602	-0.2594	0.6013	3.7168	-0.0509
Lingual_R	0.0118	0.0745	0.8057	0.2140	1.4282	0.1580		0.0051	0.0704	0.3721	0.3565	2.7705	0.0730
Olfactory_R	0.0117	0.0563	1.0596	0.1497	1.0826	0.2078		0.0149	0.0636	1.1979	0.1211	1.6461	0.2349
Lingual_L	0.0103	0.0656	0.8007	0.2154	1.4282	0.1570		-0.0192	0.0553	-1.7725	0.9558	4.9681	-0.3476
Occipital_Inf_L	0.0102	0.0737	0.7070	0.2430	1.5882	0.1387		0.0019	0.0672	0.1468	0.4422	3.2111	0.0288
Pallidum_R	0.0095	0.0870	0.5547	0.2920	1.8299	0.1088		0.0002	0.0833	0.0127	0.4950	3.4833	0.0025
Amygdala_R	0.0088	0.0902	0.5000	0.3107	1.8952	0.0981		0.0094	0.0738	0.6479	0.2615	2.5049	0.1271
Temporal_Pole_Mid_R	0.0083	0.0750	0.5640	0.2889	1.8299	0.1106		-0.0131	0.0629	-1.0629	0.8510	4.6342	-0.2084
Temporal_Sup_L	0.0081	0.0668	0.6201	0.2704	1.7421	0.1216		0.0084	0.0732	0.5859	0.2816	2.5258	0.1149
Occipital_Inf_R	0.0055	0.0525	0.5301	0.3004	1.8568	0.1040		-0.0006	0.0495	-0.0642	0.5254	3.5741	-0.0126
Olfactory_L	0.0044	0.0710	0.3177	0.3767	2.2132	0.0623		0.0035	0.0568	0.3145	0.3779	2.8809	0.0617
Caudate_R	0.0041	0.0582	0.3597	0.3611	2.1731	0.0705		-0.0020	0.0620	-0.1631	0.5641	3.6534	-0.0320
Temporal_Pole_Sup_L	0.0034	0.0549	0.3158	0.3774	2.2132	0.0619		-0.0095	0.0476	-1.0139	0.8398	4.6342	-0.1988
Temporal_Pole_Sup_R	0.0029	0.0570	0.2601	0.3985	2.3072	0.0510		-0.0067	0.0641	-0.5339	0.7009	4.1106	-0.1047
Caudate_L	0.0012	0.0705	0.0895	0.4647	2.6490	0.0176		-0.0046	0.0708	-0.3287	0.6274	3.8267	-0.0645
Heschl_R	0.0010	0.0655	0.0784	0.4691	2.6490	0.0154		0.0121	0.0865	0.7155	0.2405	2.4445	0.1403
Paracentral_Lobule_R	0.0007	0.0731	0.0477	0.4811	2.6841	0.0094		-0.0223	0.0717	-1.5844	0.9372	4.9388	-0.3107
Heschl_L	-0.0007	0.0632	-0.053	0.5211	2.8717	-0.011		-0.0172	0.0754	-1.1668	0.8728	4.6973	-0.2288
Insula_R	-0.0030	0.0497	-0.303	0.6178	3.2859	-0.059		-0.0131	0.0645	-1.0367	0.8451	4.6342	-0.2033
Frontal_Sup_Orb_L	-0.0037	0.0674	-0.278	0.6083	3.2736	-0.055		-0.0079	0.0559	-0.7219	0.7615	4.3541	-0.1416
Rolandic_Oper_R	-0.0038	0.0777	-0.250	0.5975	3.2539	-0.049		-0.0065	0.0923	-0.3595	0.6389	3.8454	-0.0705
Amygdala_L	-0.0049	0.0617	-0.406	0.6560	3.4491	-0.080		-0.0050	0.0576	-0.4422	0.6689	3.9739	-0.0867
Putamen_L	-0.0081	0.0744	-0.555	0.7080	3.6805	-0.109		-0.0352	0.0699	-2.5671	0.9917	5.0558	-0.5034
Thalamus_R	-0.0204	0.0782	-1.329	0.9021	4.6365	-0.261		-0.0130	0.0636	-1.0415	0.8462	4.6342	-0.2043
Putamen_R	-0.0246	0.0731	-1.718	0.9510	4.8333	-0.337		-0.0399	0.0735	-2.7646	0.9947	5.0558	-0.5422

Supplementary Table 2. Complete listing of ‘individual-differences” analyses for all ROIs, serving as a companion to **Figures 3 and 6**. The leftmost (second to seventh) columns cover partial RSA results, when each individual’s fMRI data was compared to their own personal multimodal model, when controlling for a group-average multimodal model derived from the other participants (as in **Figure 3**). t>0 and p correspond to the t-statistic and uncorrected p-value associated with one sample t-tests against zero (1-tailed). fdrP corresponds to False Discovery Rate (FDR) corrected p-values. d corresponds to Cohen’s d, which was computed by dividing the t-statistic by $26^{1/2}$. The rightmost three columns list results arising from decoding tests when using individual’s personal models to identify their fMRI data (as in **Figure 6**). decode_acc indicates the proportion of times (0 to 1) that individuals were correctly identified (Note that these values were transformed to percentages in **Figure 6**). decode_p indicates the associated p-values computed using permutation testing. Note that the p-values tabulated below are not precisely the same as **Figure 6**, because **Figure 6** was computed with a permutation test on a different random seed. decode_fdrP corresponds to the FDR corrected decode_p value. Rows shaded in grey correspond to the 8 ROIs identified in **Supplementary Table 1**/**Figure 4**). FDR correction in the below table was computed across these 8 ROIs.

ROI	mean(rPrtOwnMdlCntrGrp)	SD(rPrtOwnMdlCntrlGrp)	t>0	p	fdrP	d		decode_acc	decode_p	decode_fdrP
Precuneus_L	0.0780	0.0975	4.0785	0.0002	0.0030	0.7999		0.7846	0.0002	0.0033
Precuneus_R	0.0805	0.1081	3.7944	0.0004	0.0030	0.7441		0.7662	0.0006	0.0043
Temporal_Mid_L	0.0643	0.0931	3.5204	0.0008	0.0034	0.6904		0.7754	0.0003	0.0033
Parietal_Inf_L	0.0583	0.0856	3.4735	0.0009	0.0034	0.6812		0.7231	0.0016	0.0058
Cingulum_Post_L	0.0575	0.0830	3.5344	0.0008	0.0034	0.6932		0.7477	0.0008	0.0043
Temporal_Mid_R	0.0681	0.1017	3.4145	0.0011	NaN	0.6696		0.7631	0.0003	NaN
Occipital_Mid_L	0.0502	0.0830	3.0833	0.0025	0.0077	0.6047		0.6862	0.0103	0.0320
Frontal_Mid_L	0.0620	0.0829	3.8139	0.0004	0.0030	0.7480		0.7415	0.0010	0.0043
Angular_L	0.0425	0.0939	2.3104	0.0147	0.0400	0.4531		0.6492	0.0326	0.0886
Frontal_Sup_L	0.0523	0.0767	3.4740	0.0009	NaN	0.6813		0.7600	0.0004	NaN
Angular_R	0.0478	0.0916	2.6607	0.0067	NaN	0.5218		0.6985	0.0074	NaN
Frontal_Med_Orb_L	0.0456	0.0593	3.9278	0.0003	NaN	0.7703		0.7662	0.0002	NaN
Frontal_Sup_Medial_L	0.0419	0.0716	2.9806	0.0032	NaN	0.5846		0.7415	0.0007	NaN
Cingulum_Post_R	0.0369	0.0873	2.1531	0.0206	NaN	0.4223		0.6646	0.0194	NaN
Frontal_Inf_Orb_L	0.0520	0.0664	3.9973	0.0002	NaN	0.7839		0.7815	0.0004	NaN
Frontal_Sup_Medial_R	0.0463	0.0686	3.4399	0.0010	NaN	0.6746		0.7631	0.0002	NaN
Parietal_Sup_L	0.0371	0.0810	2.3360	0.0139	NaN	0.4581		0.6769	0.0129	NaN
Frontal_Inf_Tri_L	0.0324	0.0711	2.3280	0.0142	NaN	0.4566		0.6769	0.0108	NaN
Cuneus_L	0.0526	0.0735	3.6473	0.0006	NaN	0.7153		0.7692	0.0005	NaN
Frontal_Mid_R	0.0494	0.0665	3.7849	0.0004	NaN	0.7423		0.7569	0.0005	NaN
Precentral_L	0.0458	0.0744	3.1388	0.0022	NaN	0.6156		0.7262	0.0019	NaN
SupraMarginal_L	0.0303	0.0939	1.6460	0.0561	NaN	0.3228		0.6000	0.1067	NaN
Frontal_Sup_R	0.0326	0.0874	1.9013	0.0344	NaN	0.3729		0.6554	0.0256	NaN
Temporal_Inf_L	0.0371	0.0844	2.2401	0.0171	NaN	0.4393		0.6831	0.0108	NaN
Occipital_Mid_R	0.0378	0.0848	2.2765	0.0158	NaN	0.4465		0.6646	0.0222	NaN
Cingulum_Mid_L	0.0178	0.0723	1.2540	0.1107	NaN	0.2459		0.6185	0.0708	NaN
Parietal_Sup_R	0.0325	0.0730	2.2726	0.0160	NaN	0.4457		0.6677	0.0167	NaN
Cingulum_Ant_L	0.0377	0.0772	2.4905	0.0099	NaN	0.4884		0.7231	0.0020	NaN
Rectus_L	0.0302	0.0738	2.0861	0.0237	NaN	0.4091		0.6862	0.0109	NaN
Frontal_Inf_Orb_R	0.0329	0.0725	2.3120	0.0147	NaN	0.4534		0.7169	0.0034	NaN
Supp_Motor_Area_L	0.0374	0.0719	2.6522	0.0068	NaN	0.5201		0.6892	0.0092	NaN
ParaHippocampal_L	0.0371	0.0687	2.7578	0.0054	NaN	0.5408		0.6892	0.0088	NaN
Frontal_Mid_Orb_L	0.0281	0.0676	2.1193	0.0221	NaN	0.4156		0.6769	0.0144	NaN
Calcarine_L	0.0236	0.0688	1.7492	0.0463	NaN	0.3430		0.6062	0.0937	NaN
Hippocampus_R	0.0288	0.0848	1.7345	0.0476	NaN	0.3402		0.6431	0.0376	NaN
Hippocampus_L	0.0326	0.0559	2.9745	0.0032	NaN	0.5834		0.6800	0.0131	NaN
Frontal_Mid_Orb_R	0.0367	0.0761	2.4620	0.0105	NaN	0.4828		0.6831	0.0117	NaN
Temporal_Pole_Mid_L	0.0211	0.0740	1.4531	0.0793	NaN	0.2850		0.6000	0.1077	NaN
Postcentral_L	0.0210	0.0678	1.5786	0.0635	NaN	0.3096		0.5908	0.1328	NaN

Frontal_Inf_Oper_L	0.0138	0.0560	1.2527	0.1110	NaN	0.2457		0.5908	0.1249	NaN
Supp_Motor_Area_R	0.0233	0.0938	1.2654	0.1087	NaN	0.2482		0.6000	0.1018	NaN
Calcarine_R	0.0250	0.0835	1.5255	0.0698	NaN	0.2992		0.6585	0.0241	NaN
Precentral_R	0.0294	0.0722	2.0808	0.0239	NaN	0.4081		0.6492	0.0324	NaN
ParaHippocampal_R	0.0273	0.0735	1.8904	0.0352	NaN	0.3707		0.6523	0.0287	NaN
Occipital_Sup_R	0.0215	0.0599	1.8314	0.0395	NaN	0.3592		0.6215	0.0699	NaN
Fusiform_R	0.0249	0.0754	1.6809	0.0526	NaN	0.3297		0.6277	0.0589	NaN
Parietal_Inf_R	0.0165	0.0670	1.2544	0.1106	NaN	0.2460		0.6092	0.0934	NaN
Temporal_Inf_R	0.0366	0.0890	2.0982	0.0231	NaN	0.4115		0.7138	0.0033	NaN
Temporal_Sup_R	0.0144	0.0758	0.9699	0.1707	NaN	0.1902		0.5600	0.2251	NaN
Cingulum_Mid_R	0.0228	0.0915	1.2722	0.1075	NaN	0.2495		0.5938	0.1271	NaN
SupraMarginal_R	0.0082	0.0973	0.4273	0.3364	NaN	0.0838		0.5446	0.2841	NaN
Occipital_Sup_L	0.0279	0.0678	2.0951	0.0232	NaN	0.4109		0.6400	0.0369	NaN
Thalamus_L	0.0253	0.0772	1.6720	0.0535	NaN	0.3279		0.6154	0.0767	NaN
Frontal_Med_Orb_R	0.0105	0.0651	0.8223	0.2093	NaN	0.1613		0.5815	0.1524	NaN
Rolandic_Oper_L	0.0149	0.0826	0.9202	0.1831	NaN	0.1805		0.5723	0.1827	NaN
Rectus_R	0.0191	0.0759	1.2835	0.1055	NaN	0.2517		0.5815	0.1623	NaN
Cingulum_Ant_R	0.0131	0.0721	0.9282	0.1811	NaN	0.1820		0.5569	0.2399	NaN
Pallidum_L	0.0357	0.0883	2.0631	0.0248	NaN	0.4046		0.7108	0.0044	NaN
Frontal_Inf_Tri_R	0.0201	0.0655	1.5634	0.0653	NaN	0.3066		0.6338	0.0488	NaN
Postcentral_R	0.0101	0.0763	0.6724	0.2537	NaN	0.1319		0.5231	0.3861	NaN
Fusiform_L	0.0175	0.0645	1.3819	0.0896	NaN	0.2710		0.6123	0.0817	NaN
Frontal_Sup_Orb_R	0.0198	0.0596	1.6960	0.0512	NaN	0.3326		0.5877	0.1382	NaN
Insula_L	0.0122	0.0721	0.8664	0.1973	NaN	0.1699		0.5415	0.2910	NaN
Cuneus_R	0.0096	0.0653	0.7500	0.2301	NaN	0.1471		0.5446	0.2841	NaN
Frontal_Inf_Oper_R	0.0155	0.0643	1.2261	0.1158	NaN	0.2405		0.6031	0.1048	NaN
Paracentral_Lobule_L	0.0157	0.0895	0.8917	0.1905	NaN	0.1749		0.5631	0.2142	NaN
Lingual_R	0.0119	0.0681	0.8907	0.1908	NaN	0.1747		0.5508	0.2593	NaN
Olfactory_R	0.0006	0.0603	0.0489	0.4807	NaN	0.0096		0.5015	0.4864	NaN
Lingual_L	0.0257	0.0659	1.9840	0.0292	NaN	0.3891		0.6400	0.0404	NaN
Occipital_Inf_L	0.0120	0.0621	0.9855	0.1669	NaN	0.1933		0.5354	0.3277	NaN
Pallidum_R	0.0079	0.0994	0.4031	0.3451	NaN	0.0791		0.5692	0.1987	NaN
Amygdala_R	0.0008	0.0868	0.0482	0.4810	NaN	0.0095		0.5508	0.2589	NaN
Temporal_Pole_Mid_R	0.0171	0.0675	1.2894	0.1045	NaN	0.2529		0.6062	0.0936	NaN
Temporal_Sup_L	0.0016	0.0577	0.1394	0.4451	NaN	0.0273		0.5077	0.4563	NaN
Occipital_Inf_R	0.0101	0.0703	0.7356	0.2344	NaN	0.1443		0.5262	0.3683	NaN
Olfactory_L	0.0006	0.0733	0.0436	0.4828	NaN	0.0086		0.5046	0.4667	NaN
Caudate_R	0.0062	0.0688	0.4605	0.3246	NaN	0.0903		0.5662	0.2058	NaN
Temporal_Pole_Sup_L	0.0073	0.0659	0.5631	0.2892	NaN	0.1104		0.5477	0.2773	NaN
Temporal_Pole_Sup_R	0.0038	0.0527	0.3671	0.3583	NaN	0.0720		0.5538	0.2534	NaN
Caudate_L	0.0056	0.0726	0.3937	0.3486	NaN	0.0772		0.5354	0.3231	NaN
Heschl_R	-0.0092	0.0676	-0.6949	0.7532	NaN	-0.1363		0.4677	0.6449	NaN
Paracentral_Lobule_R	0.0123	0.0754	0.8350	0.2058	NaN	0.1638		0.5846	0.1490	NaN
Heschl_L	0.0076	0.0748	0.5193	0.3041	NaN	0.1018		0.5446	0.2786	NaN
Insula_R	0.0029	0.0462	0.3219	0.3751	NaN	0.0631		0.4954	0.5135	NaN
Frontal_Sup_Orb_L	0.0001	0.0655	0.0105	0.4959	NaN	0.0021		0.5200	0.4019	NaN
Rolandic_Oper_R	-0.0007	0.0666	-0.0560	0.5221	NaN	-0.0110		0.4554	0.7004	NaN
Amygdala_L	-0.0031	0.0673	-0.2356	0.5922	NaN	-0.0462		0.4923	0.5260	NaN
Putamen_L	0.0136	0.0701	0.9883	0.1662	NaN	0.1938		0.5477	0.2819	NaN
Thalamus_R	-0.0165	0.0862	-0.9757	0.8307	NaN	-0.1914		0.4462	0.7276	NaN
Putamen_R	-0.0011	0.0671	-0.0842	0.5332	NaN	-0.0165		0.4708	0.6329	NaN

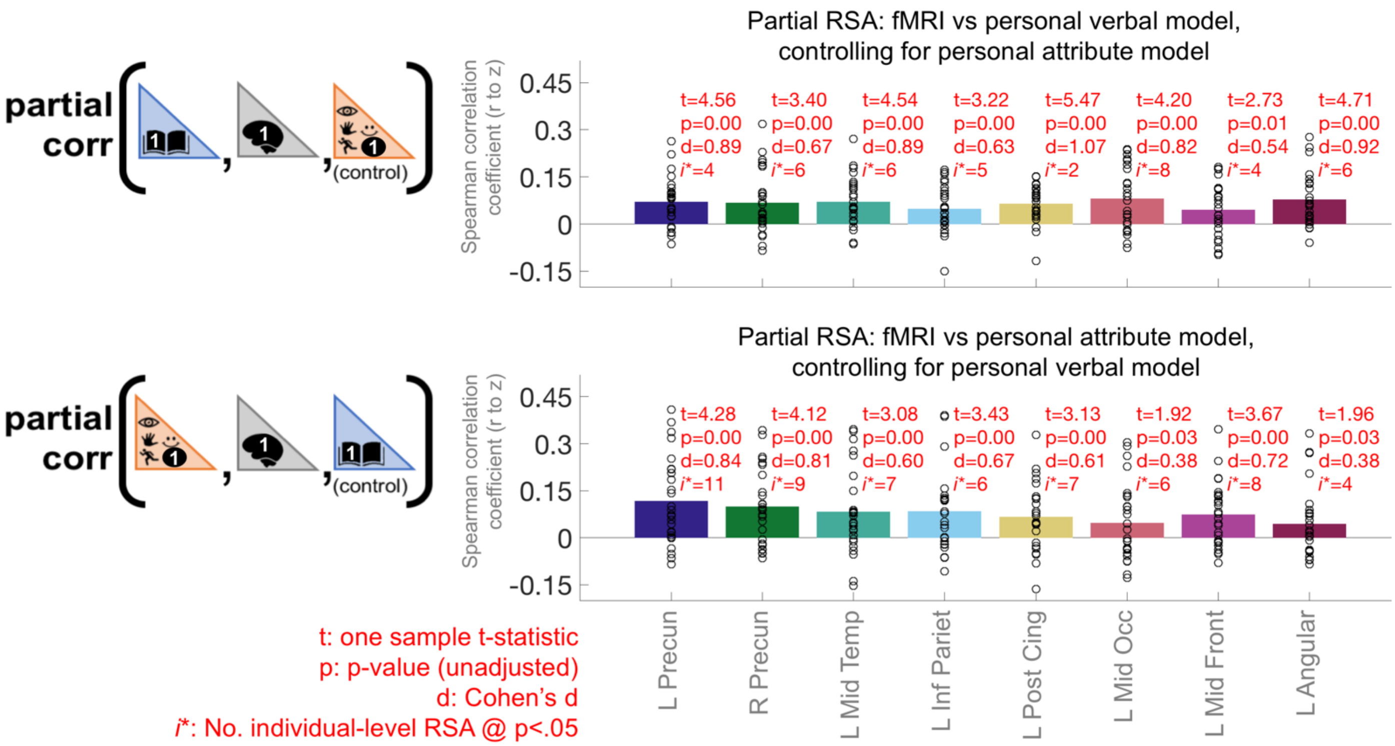

Supplementary Figure 2. Both the verbal and attribute models contributed to predicting person-specific fMRI representations. We estimated whether both the verbal and attribute models had made independent contributions to predicting fMRI representational structure in the eight anatomical ROIs illustrated in **Figure 3**. To test this, we ran a partial correlation-based RSA: fMRI similarity vectors were correlated with verbal similarity vectors whilst controlling for attribute similarity vectors and vice versa. In both cases similarity vectors were person-specific. Partial correlation coefficients for each participant were r-to-z transformed. One sample t-tests were then applied to test whether the set of values was greater than zero (1-tailed). The bar plots illustrate the results for each of the 8 ROIs identified in **Figure 3.** Open black circles illustrate Partial RSA coefficients for each of the 26 participants. Bar heights correspond to the mean value across participants. t and p are the outcomes of the t-tests. P-values are uncorrected for multiple comparisons. Exact p-values for the verbal model in the same order as plotted above were: 0.0001, 0.0011, 0.0001, 0.0018, 0.0000, 0.0001, 0.0057, 0.0000. Exact p-values for the attribute model were: 0.0001, 0.0002, 0.0025, 0.0011, 0.0022, 0.0334, 0.0006, 0.0309. Cohen's d was computed by dividing the t-statistic by $26^{1/2}$ (corresponding to 26 participants). *i** identifies the number of individual-level RSA permutation p-values < 0.05 (maximum 26, see **Methods** for details). In all eight ROIs partial correlation coefficients associated with both the verbal and attribute models were significantly greater than zero. Critically these results provided evidence that both the verbal and attribute models played complementary roles in explaining individual differences in brain activity. To provide extra context for the illustrated RSA coefficients, we computed RSA between person-specific verbal and attribute models within each participant. This yielded a mean±SEM Spearman correlation coefficient of 0.23±0.02 across the 26 participants, which was significantly greater than zero (t=13.419, d=2.6, p=3e-13, one sample t-test) reflecting a strong overlap in information content across verbal/attribute models. **Supplementary Figure 16 and 17** illustrate comparative results using 50 and 200 voxels per ROI. Source data are provided as a Source Data file.

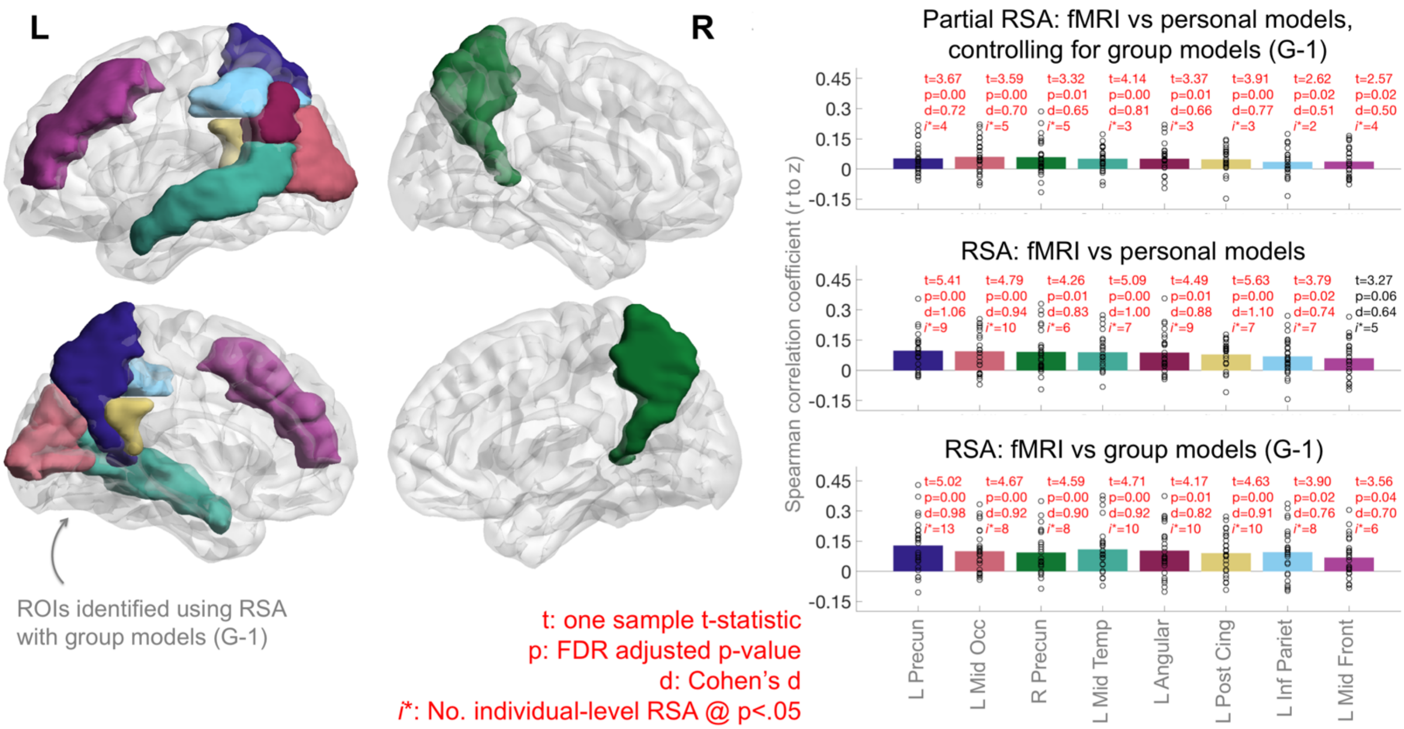

Supplementary Figure 3. Replication of Figure 3 "fMRI activation patterns elicited in imagining common scenarios reflect person-specific information" and Figure 4 when *differently* analyses were computed using the Verbal models only (rather than Multimodal models). The entire analysis presented in **Figure 3 and 4** was repeated from scratch and yielded a broadly similar patterns of results. Open black circles illustrate RSA coefficients for the 26 participants. Bar heights correspond to mean values across participants. One sample t-tests tested whether RSA coefficients were greater than zero (1-tail). Cohen's d was computed by dividing the t-statistic by $26^{1/2}$. *i** identifies the number of individual-level RSA permutation p-values < 0.05 (maximum 26, see **Methods** for details). The mid-right and bottom right plots illustrate RSA comparisons of fMRI data with person-specific models and group-average models respectively. The eight ROIs presented were selected according to the procedure described in **Methods**. FDR correction of p-values in the partial RSA (top) was across eight ROIs. The exact FDR corrected p-values in the same order as plotted above were: 0.0038, 0.0038, 0.0051, 0.0034, 0.0051, 0.0034, 0.0222, 0.0222. FDR[64] correction of p-values for person-specific and group-average models (mid and bottom plot) was across 90 ROIs. Exact FDR corrected p-values for person-specific models were: 0.0015, 0.0036, 0.0084, 0.0022, 0.0056, 0.0015, 0.0245, 0.0602. Exact FDR corrected p-values for group-average models were: 0.0050, 0.0050, 0.0050, 0.0050, 0.0122, 0.0050, 0.0209, 0.0435. Brain illustrations were made using Ref[65]. Source data are provided as a Source Data file.

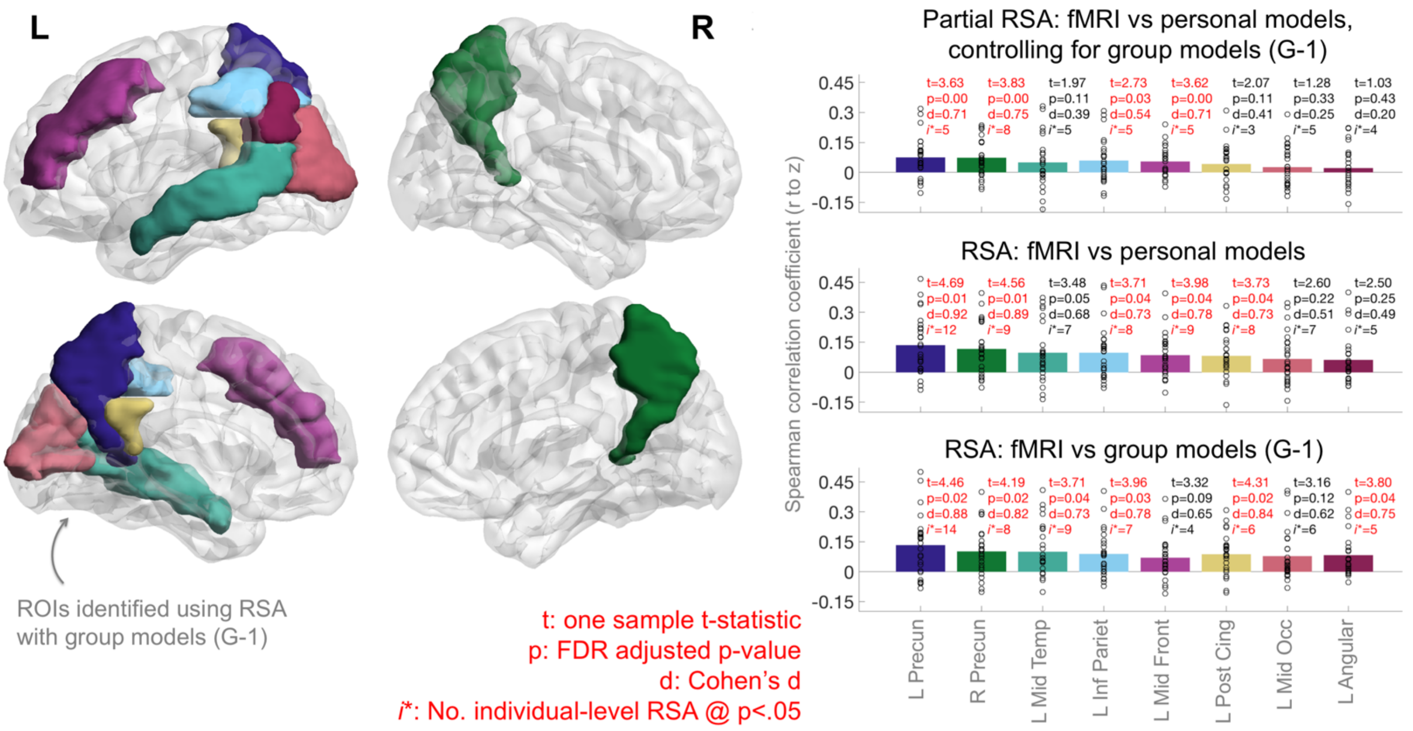

Supplementary Figure 4. Replication of Figure 3 "fMRI activation patterns elicited in imagining common scenarios reflect person-specific information" and Figure 4 when *differently* analyses were computed using the Attribute models only (rather than Multimodal models). The entire analysis presented in **Figure 3 and 4** was repeated from scratch and yielded a broadly similar patterns of results. However, significant partial correlation coefficients (top right) were obtained from four ROIs. Open black circles illustrate RSA coefficients for the 26 participants. Bar heights correspond to mean values across participants. One sample t-tests tested whether RSA coefficients were greater than zero (1-tail). Cohen's d was computed by dividing the t-statistic by $26^{1/2}$. *i** identifies the number of individual-level RSA permutation p-values < 0.05 (maximum 26, see **Methods** for details). The mid-right and bottom right plots illustrate RSA comparisons of fMRI data with person-specific models and group-average models respectively. The eight ROIs presented were selected according to the procedure described in **Methods**. FDR[64] correction of p-values for person-specific and group-average models (mid and bottom plot) was across 90 ROIs. FDR correction of p-values in the partial RSA (top) was across eight ROIs. The exact FDR corrected p-values in the same order as plotted above were: 0.0047, 0.0047, 0.1092, 0.0308, 0.0047, 0.1063, 0.3322, 0.4269. FDR correction of p-values for person-specific and group-average models (mid and bottom plot) was across 90 ROIs. Exact FDR corrected p-values for person-specific models were: 0.0133, 0.0133, 0.0532, 0.0408, 0.0399, 0.0408, 0.2248, 0.2465. Exact FDR corrected p-values for group-average models were: 0.0233, 0.0233, 0.0391, 0.0316, 0.0908, 0.0233, 0.1170, 0.0377. Brain illustrations were made using Ref[65]. Source data are provided as a Source Data file.

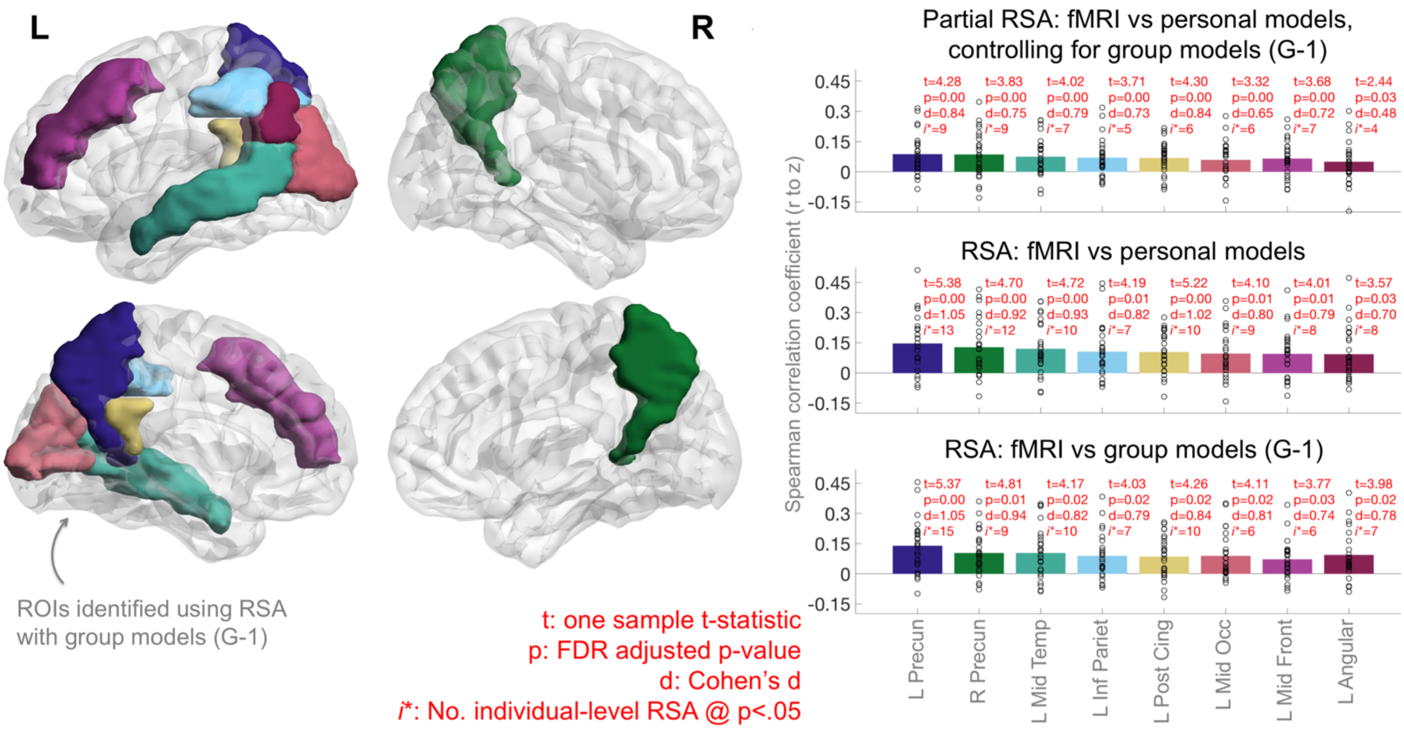

Supplementary Figure 5. Replication of Figure 3 "fMRI activation patterns elicited in imagining common scenarios reflect person-specific information" and Figure 4 when *differently* group-average model representations were computed by taking the pointwise mean of personal models in model feature space (and then computing a group-level similarity matrix from this averaged data rather than averaging personal similarity matrices as was illustrated in **Figure 2**). The entire analysis presented in **Figure 3 and 4** was repeated from scratch using this alternative group-averaging strategy, and yielded broadly the same pattern of results. Open black circles illustrate RSA coefficients for the 26 participants. Bar heights correspond to mean values across participants. One sample t-tests tested whether RSA coefficients were greater than zero (1-tail). Cohen's d was computed by dividing the t-statistic by $26^{1/2}$. *i** identifies the number of individual-level RSA permutation p-values < 0.05 (maximum 26, see **Methods** for details). The mid-right and bottom right plots illustrate RSA comparisons of fMRI data with person-specific models and group-average models respectively. The eight ROIs presented were selected according to the procedure described in **Methods**. FDR[64] correction of p-values in the partial RSA (top) was across eight ROIs. The exact FDR corrected p-values in the same order as plotted above were: 0.0013, 0.0020, 0.0017, 0.0020, 0.0013, 0.0043, 0.0020, 0.0304. FDR correction of p-values for person-specific and group-average models (mid and bottom plot) was across 90 ROIs. Exact FDR corrected p-values for person-specific models were: 0.0024, 0.0047, 0.0047, 0.0116, 0.0024, 0.0124, 0.0138, 0.0327. Exact FDR corrected p-values for group-average models were: 0.0033, 0.0069, 0.0169, 0.0170, 0.0169, 0.0169, 0.0256, 0.0170. Brain illustrations were made using Ref[65]. Source data are provided as a Source Data file.

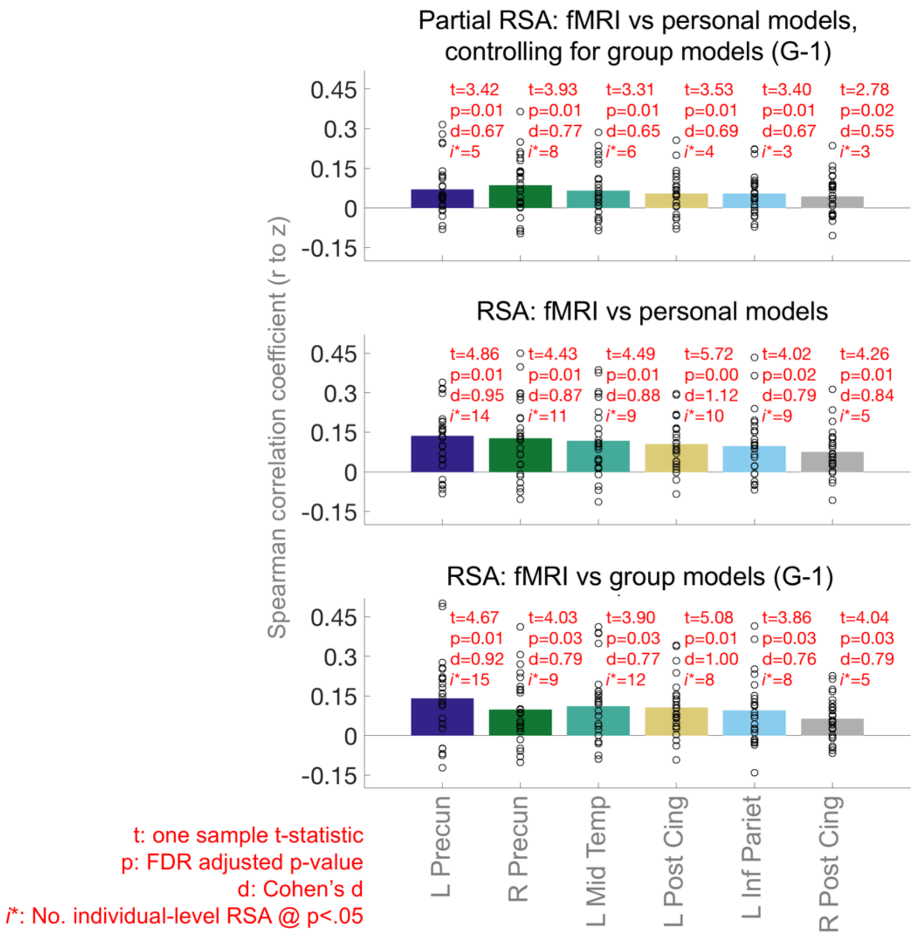

Supplementary Figure 6. Replication of Figure 3 "fMRI activation patterns elicited in imagining common scenarios reflect person-specific information" and Figure 4 when *differently* the analyses were performed on 50 rather than 100 voxels per ROI. The entire analysis presented in **Figure 3 and 4** was repeated from scratch and yielded a broadly similar pattern of results, albeit with 6 ROIs passing FDR corrected statistical significance thresholds when using group average models (bottom right). Of these 6 ROIs, Right Posterior Cingulate cortex (grey bars) had not been identified in **Figures 3 and 4**. The other five ROIs were the same as **Figures 3 and 4**. Open black circles illustrate RSA coefficients for the 26 participants. Bar heights correspond to mean values across participants. One sample t-tests tested whether RSA coefficients were greater than zero. Cohen's d was computed by dividing the t-statistic by $26^{1/2}$. *i** identifies the number of individual-level RSA permutation p-values < 0.05 (maximum 26, see **Methods** for details). The mid-right and bottom right plots illustrate RSA comparisons of fMRI data with person-specific models and group-average models respectively. The six ROIs presented were selected according to the procedure described in **Methods**. FDR[64] correction of p-values in the partial RSA (top) was across six ROIs. The exact FDR corrected p-values in the same order as plotted above were: 0.0051, 0.0051, 0.0052, 0.0051, 0.0051, 0.0153. FDR correction of p-values for person-specific and group-average models (mid and bottom plot) was across 90 ROIs. Exact FDR corrected p-values for person-specific models were: 0.0062, 0.0094, 0.0094, 0.0014, 0.0155, 0.0114. Exact FDR corrected p-values for group-average models were: 0.0101, 0.0262, 0.0272, 0.0069, 0.0272, 0.0262, 0.0327. Brain illustrations were made using Ref[65]. Source data are provided as a Source Data file.

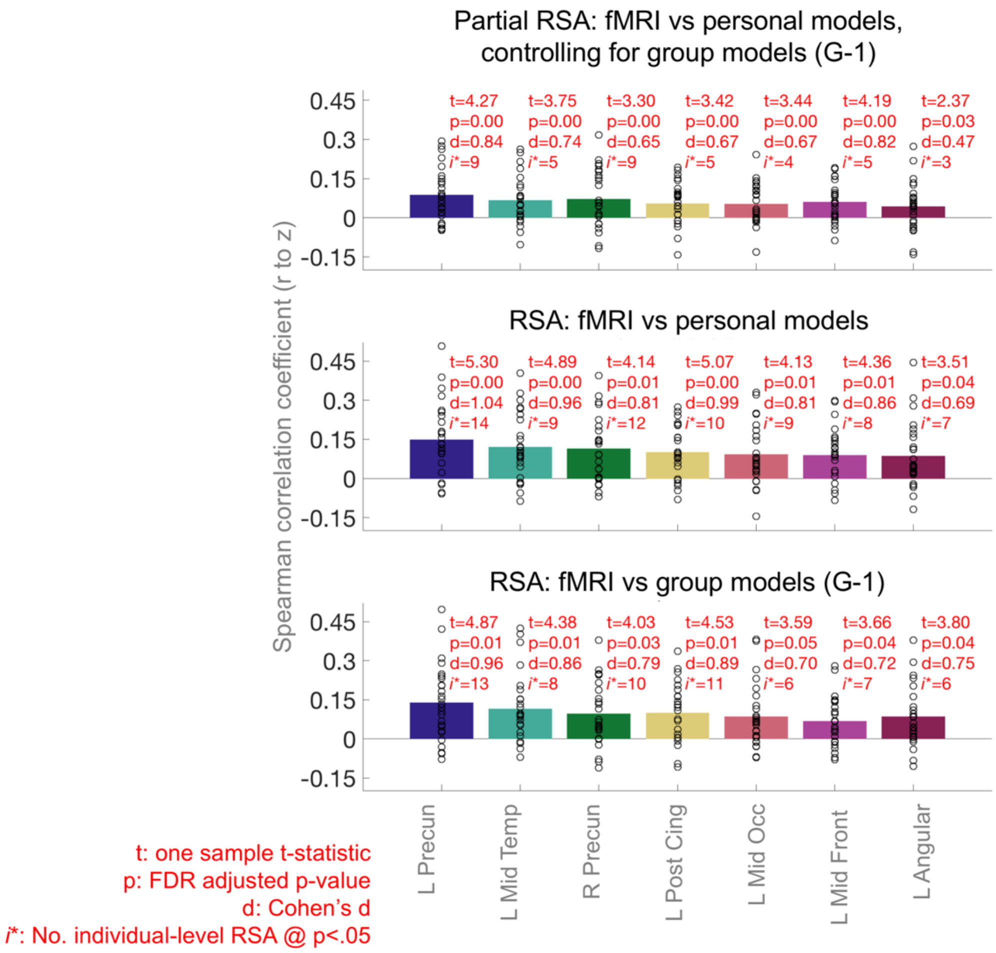

Supplementary Figure 7. Replication of Figure 3 "fMRI activation patterns elicited in imagining common scenarios reflect person-specific information" and Figure 4 when *differently* the analyses were performed on 200 rather than 100 voxels per ROI. The entire analysis presented in **Figure 3 and 4** was repeated from scratch and yielded a broadly similar patterns of results albeit with 7 ROIs passing FDR corrected statistical significance thresholds when using group average models (bottom right). Open black circles illustrate RSA coefficients for the 26 participants. Bar heights correspond to mean values across participants. One sample t-tests tested whether RSA coefficients were greater than zero (1-tail). Cohen's d was computed by dividing the t-statistic by $26^{1/2}$. *i** identifies the number of individual-level RSA permutation p-values < 0.05 (maximum 26, see **Methods** for details). The mid-right and bottom right plots illustrate RSA comparisons of fMRI data with person-specific models and group-average models respectively. The seven ROIs presented were selected according to the procedure described in **Methods**. FDR correction of p-values in the partial RSA (top) was across seven ROIs. The exact FDR corrected p-values in the same order as plotted above were: 0.0014, 0.0028, 0.0044, 0.0039, 0.0039, 0.0014, 0.0334. FDR[64] correction of p-values for person-specific and group-average models (mid and bottom plot) was across 90 ROIs. Exact FDR corrected p-values for person-specific models were: 0.0030, 0.0030, 0.0117, 0.0030, 0.0117, 0.0089, 0.0368. Exact FDR corrected p-values for group-average models were: 0.0118, 0.0142, 0.0263, 0.0142, 0.0461, 0.0450, 0.0373. Brain illustrations were made using Ref[65]. Source data are provided as a Source Data file.

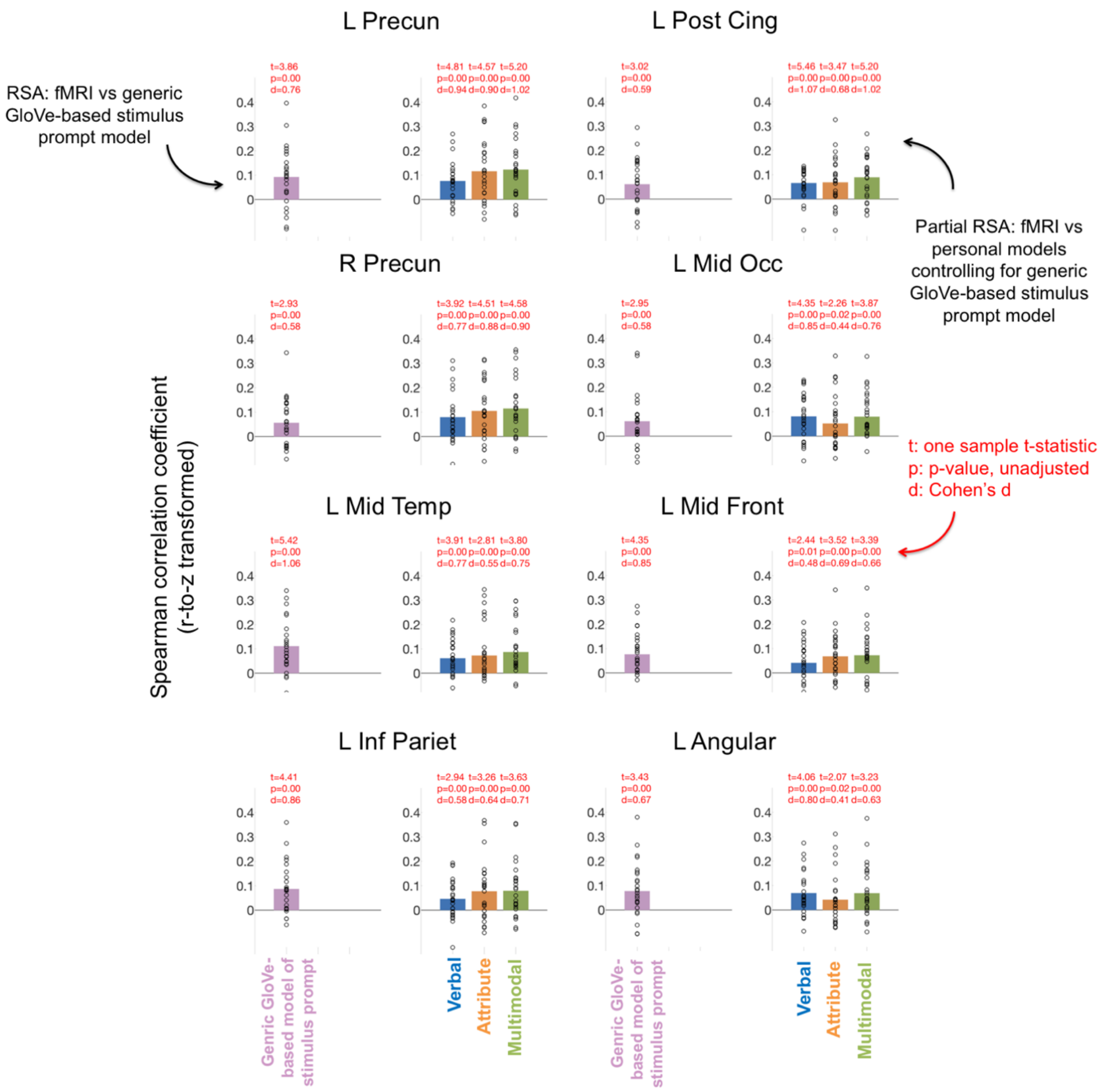

Supplementary Figure 8. Comparative RSA results to Figure 3 "fMRI activation patterns elicited in imagining common scenarios reflect person-specific information" when participants' fMRI data was compared to a generic distributional semantic model representations (GloVe) of the words in the stimulus prompt. The stimulus prompt model was built by summing GloVe vectors for content words in the stimuli e.g. "A dancing scenario" = "dancing"+"scenario" in the same way as was illustrated in **Figure 1 part 3**. Bars are Mean±SEM. One sample t-tests tested whether RSA coefficients were greater than zero (1-tail). There were 26 participants. Cohen's d was computed by dividing the t-statistic by $26^{1/2}$ (corresponding to the 26 participants). P-values were uncorrected. Exact values in order of display were: L Precun: 0.0004, 0.0000, 0.0001, 0.0000. R Precun: 0.0035, 0.0003, 0.0001, 0.0001. L Mid Temp: 0.0000, 0.0003, 0.0048, 0.0004. L Inf Pariet: 0.0001, 0.0035, 0.0016, 0.0006. L Post Cing: 0.0028, 0.0000, 0.0010, 0.0000. L Mid Occ: 0.0034, 0.0001, 0.0163, 0.0003. L Mid Front: 0.0001, 0.0110, 0.0008, 0.0012. L Angular: 0.0010, 0.0002, 0.0245, 0.0017. Source data are provided as a Source Data file.

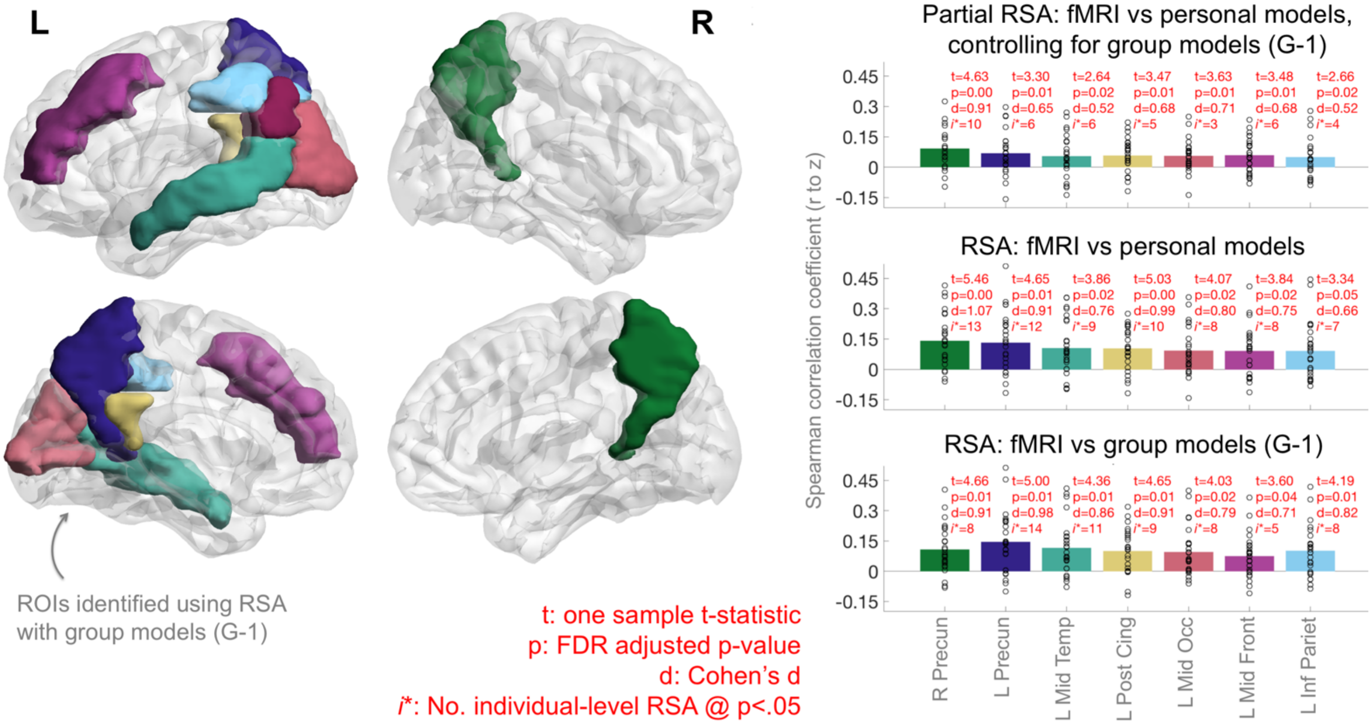

Supplementary Figure 9. Replication of Figure 3 "fMRI activation patterns elicited in imagining common scenarios reflect person-specific information" and Figure 4 when *differently* ROIs in left and right brain hemispheres of the five left handed participants brains were swapped prior to analyses to counteract possible effects surrounding lateralized language function. The entire analysis presented in **Figures 3 and 4** was repeated from scratch when the left-handers' fMRI data was flipped, and broadly the same pattern of results emerged. Open black circles illustrate RSA coefficients for the 26 participants. Bar heights correspond to mean values across participants. One sample t-tests tested whether RSA coefficients were greater than zero (1-tail). Cohen's d was computed by dividing the t-statistic by $26^{1/2}$. *i** identifies the number of individual-level RSA permutation p-values < 0.05 (maximum 26, see **Methods** for details). The mid-right and bottom right plots illustrate RSA comparisons of fMRI data with person-specific models and group-average models respectively. The seven ROIs presented were selected according to the procedure described in **Methods**. FDR[64] correction of p-values in the partial RSA (top) was across seven ROIs. The exact FDR corrected p-values in the same order as plotted above were: 0.0011, 0.0063, 0.0221, 0.0052, 0.0052, 0.0052, 0.0221. FDR correction of p-values for person-specific and group-average models (mid and bottom plot) was across 90 ROIs. Exact FDR corrected p-values for person-specific models were: 0.0026, 0.0071, 0.0247, 0.0039, 0.0189, 0.0247, 0.0462. Exact FDR corrected p-values for group-average models were: 0.0070, 0.0070, 0.0111, 0.0070, 0.0150, 0.0393, 0.0140. Brain illustrations were made using Ref[65]. Source data are provided as a Source Data file.

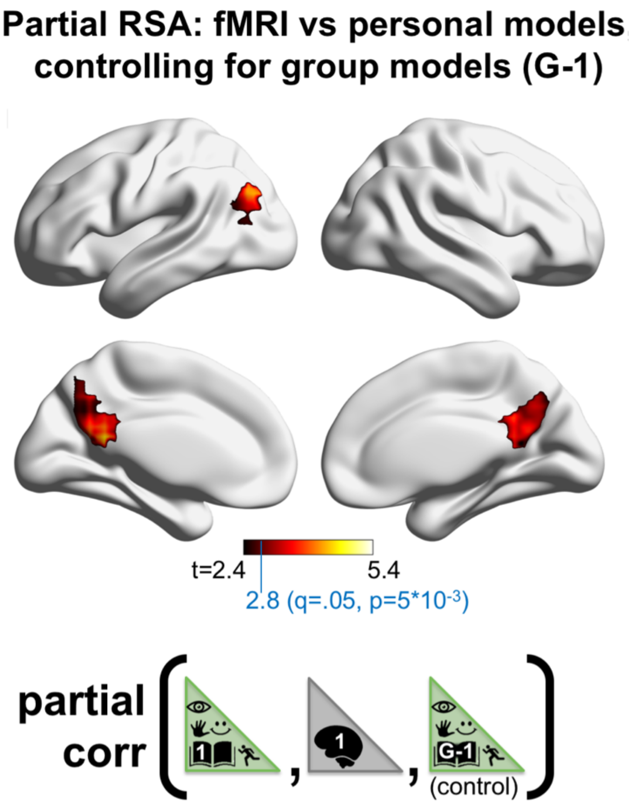

Supplementary Table 3. Companion data to Figure 5 "Neuroanatomical distribution of person-specific representational structure (RSA-Searchlight)". Listing of significant clusters for Searchlight Partial RSA: fMRI vs Personal Models controlling for Group models. Tables indicate the breakdown of ROIs contributing to significant clusters, and the x, y, z coordinates of cluster maxima (one sample t-statistic, 1-tail) in MNI space.

CLUSTER 1 NVox=449 **Left / Right Medial Parietal Cortex**

ROI	% of ROI	Max t	x	y	z
**Precuneus_L**	34.966592	4.551089	-6	-48	15
**Precuneus_R**	33.853007	3.788893	6	-57	21
**Cingulum_Post_L**	16.926503	4.716888	-6	-48	18
**Cingulum_Post_R**	8.908686	3.925046	6	-48	30
**Cuneus_L**	2.004454	3.398254	-9	-63	24
**Cingulum_Mid_R**	1.336303	3.311487	6	-51	33
**Cingulum_Mid_L**	1.113586	3.884677	0	-48	33
**Calcarine_R**	0.890869	3.527552	3	-57	12

CLUSTER 2 NVox=67 **Left Temporoparietal Junction**

ROI	% of ROI	Max t	x	y	z
**Angular_L**	41.791045	4.493385	-45	-75	30
**Temporal_Mid_L**	40.298507	3.619987	-51	-72	21
**Occipital_Mid_L**	17.910448	5.449417	-42	-81	30

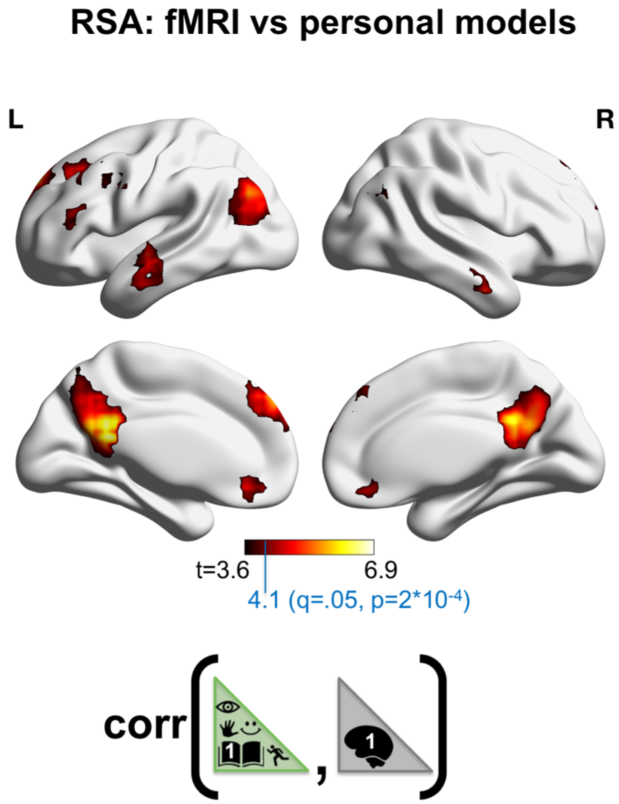

Supplementary Table 4. Companion data to Figure 5 "Neuroanatomical distribution of person-specific representational structure (RSA-Searchlight)". Significant Cluster Listing for Searchlight RSA: fMRI vs Personal Models. Tables indicate the breakdown of ROIs contributing to significant clusters, and the x, y, z coordinates of cluster maxima (one sample t-statistic, 1-tail) in MNI space.

CLUSTER: 1 NVox=1807 **Left / Right Medial Parietal Cortex / Temporoparietal Junction**

ROI	% of ROI	Max t	x	y	z
**Precuneus_L**	26.508024	6.281846	-9	-57	27
**Precuneus_R**	19.590481	5.873862	9	-51	27
**Occipital_Mid_L**	13.779745	5.95626	-42	-81	30
**Temporal_Mid_L**	6.640841	5.254832	-51	-72	21
**Cingulum_Post_L**	6.47482	6.935993	-6	-45	18
**Angular_L**	6.364139	5.754605	-45	-75	30
**Cingulum_Post_R**	4.427227	6.647541	9	-45	27
**Calcarine_L**	2.877698	4.579444	-12	-57	12
**Cuneus_L**	2.822357	5.181962	-12	-60	27
**Cingulum_Mid_L**	2.600996	5.677795	0	-48	33
**Cingulum_Mid_R**	1.936912	5.400655	9	-48	33
**Parietal_Inf_L**	1.881572	4.779021	-36	-81	39
**Calcarine_R**	1.66021	4.994735	6	-57	12
**Cuneus_R**	1.162147	4.185116	15	-66	33
**Parietal_Sup_L**	0.885445	4.302858	-27	-75	42
**Occipital_Sup_L**	0.221361	4.050967	-27	-75	39
**Lingual_L**	0.05534	3.810123	-3	-57	6
**Lingual_R**	0.05534	3.628428	6	-57	6
**Occipital_Sup_R**	0.05534	3.699634	21	-60	33

CLUSTER: 2 NVox=1215 **Left / Right Prefrontal cortex**

ROI	% of ROI	Max t	x	y	z
**Frontal_Mid_L**	25.102881	5.789302	-30	27	45
**Frontal_Sup_Medial_L**	21.893004	6.055648	-6	51	42
**Frontal_Sup_L**	16.378601	5.80208	-12	51	36
**Precentral_L**	12.098765	5.107706	-45	0	36
**Frontal_Sup_Medial_R**	10.288066	4.591245	3	54	39
**Frontal_Inf_Tri_L**	10.041152	4.685536	-42	30	12
**Frontal_Sup_R**	3.127572	4.221554	18	60	21
**Frontal_Inf_Oper_L**	0.90535	4.236608	-51	15	33
**Frontal_Mid_R**	0.164609	3.813014	21	63	27

CLUSTER: 3 NVox=256 **Left Anterior Temporal**

ROI	% of ROI	Max t	x	y	z
**Temporal_Mid_L**	83.984375	5.475016	-57	-12	-9
**Temporal_Sup_L**	14.0625	4.740621	-57	-12	-3
**Temporal_Inf_L**	1.953125	4.127434	-60	-9	-27

CLUSTER: 4 NVox=174 **Left / Right Medial Prefrontal Cortex**

ROI	% of ROI	Max t	x	y	z
**Frontal_Med_Orb_L**	33.333333	4.733286	-6	36	-12
**Frontal_Med_Orb_R**	24.137931	4.502674	3	36	-12
**Cingulum_Ant_L**	18.965517	4.582473	-6	36	-9
**Rectus_L**	12.643678	4.231672	-6	36	-18
**Rectus_R**	5.747126	3.953528	6	39	-18
**Cingulum_Ant_R**	5.172414	4.266596	3	36	-9

CLUSTER: 5 NVox=120 **Right Temporoparietal**

ROI	% of ROI	Max t	x	y	z
**Angular_R**	60	4.230188	39	-66	36
**Occipital_Mid_R**	30.833333	4.088418	36	-66	36
**Occipital_Sup_R**	5.833333	3.980142	30	-69	39
**Temporal_Mid_R**	3.333333	3.698017	48	-75	21

CLUSTER: 6 NVox=101 **Right Anterior Temporal**

ROI	% of ROI	Max t	x	y	z
**Temporal_Mid_R**	93.069307	5.388516	57	-6	-27
**Temporal_Inf_R**	6.930693	4.301431	60	-6	-30

CLUSTER: 7 NVox=22 **Left Posterior Lateral Temporal**

ROI	% of ROI	Max t	x	y	z
**Temporal_Mid_L**	90.909091	3.879143	-54	-48	0
**Temporal_Inf_L**	9.090909	3.681015	-54	-51	-6

CLUSTER: 8 NVox=7 **Right Lateral Frontal**

ROI	% of ROI	Max t	x	y	z
**Frontal_Mid_R**	57.142857	4.046126	36	0	51
**Precentral_R**	42.857143	3.860701	36	0	48

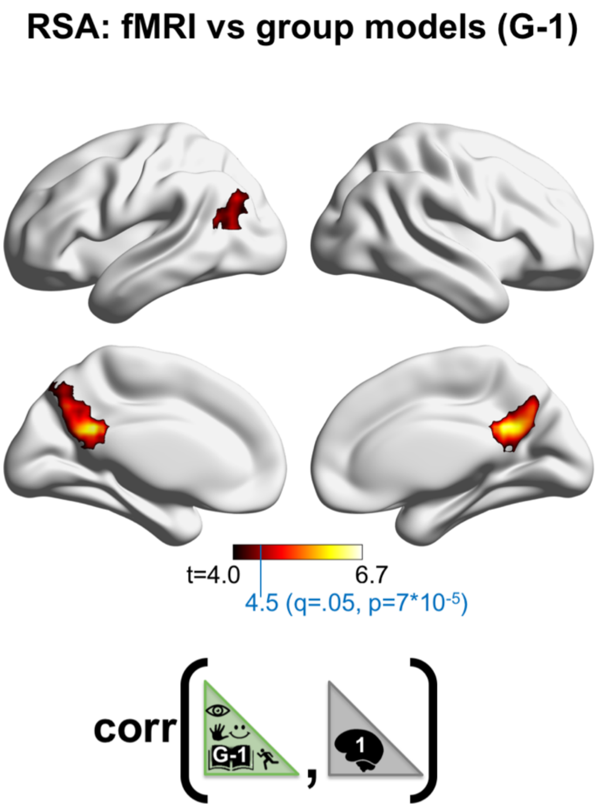

Supplementary Table 5. Companion data to Figure 5 "Neuroanatomical distribution of person-specific representational structure (RSA-Searchlight)". Significant Cluster Listing for Searchlight RSA: fMRI vs Group Models. Tables indicate the breakdown of ROIs contributing to significant clusters, and the x, y, z coordinates of cluster maxima (one sample t-statistic, 1-tail) in MNI space.

CLUSTER: 1 NVox=554 **Left / Right Medial Parietal Cortex**

ROI	% of ROI	Max t	x	y	z
**Precuneus_L**	40.613718	6.042990	-6	-54	24
**Precuneus_R**	32.310469	6.723471	9	-51	27
**Cingulum_Post_L**	14.079422	6.738582	-3	-48	27
**Cingulum_Post_R**	8.303249	6.472886	6	-48	27
**Cuneus_L**	1.624549	4.980007	0	-66	24
**Cingulum_Mid_L**	1.083032	4.999983	0	-48	33
**Cingulum_Mid_R**	1.083032	5.094678	9	-51	33
**Calcarine_R**	0.722022	4.714324	6	-57	12
**Cuneus_R**	0.180505	4.676269	15	-66	33

CLUSTER: 2 NVox=110 **Left Temporoparietal**

ROI	% of ROI	Max t	x	y	z
**Temporal_Mid_L**	58.181818	5.687535	-51	-69	18
**Angular_L**	25.454545	5.255955	-51	-69	24
**Occipital_Mid_L**	16.363636	5.200585	-51	-72	15

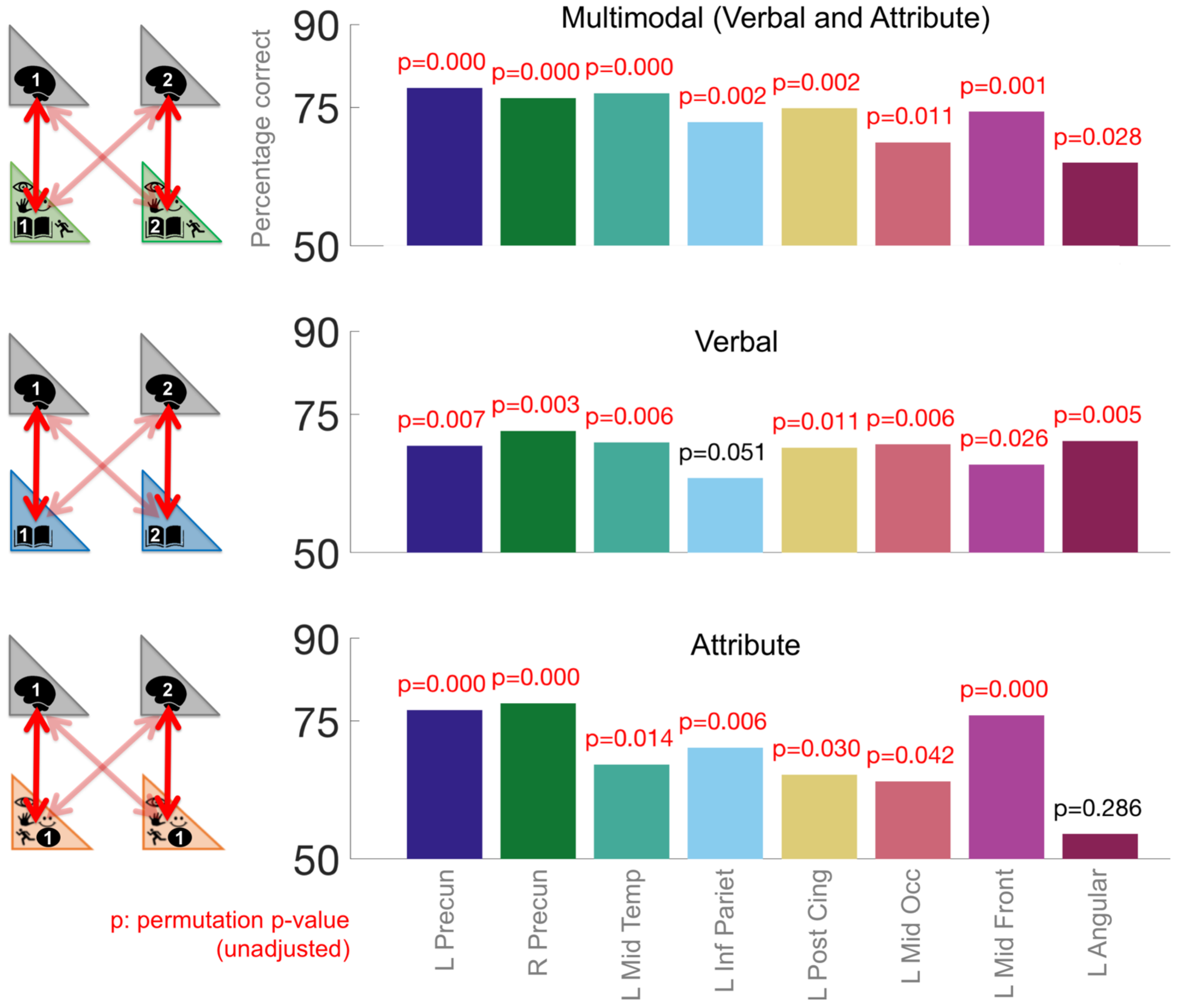

Supplementary Figure 10. Replication of Figure 6 "Individual identity can be decoded from fMRI activity elicited during the imagination of common scenarios" when the analysis was performed using verbal and attribute models in isolation. Tests were repeated for each pairwise combination of the 26 participants. Each bar illustrates the percentage of times that participant-specific models better predicted the same participant's fMRI representations than another participants' fMRI data (see **Figure 2** and main text for details). P-values were estimated using permutation tests (see **Methods**) and are uncorrected. The 8 ROIs illustrated were identified in **Figure 4**. Complete results for all ROIs are in **Supplementary Table 2.** Source data are provided as a Source Data file.

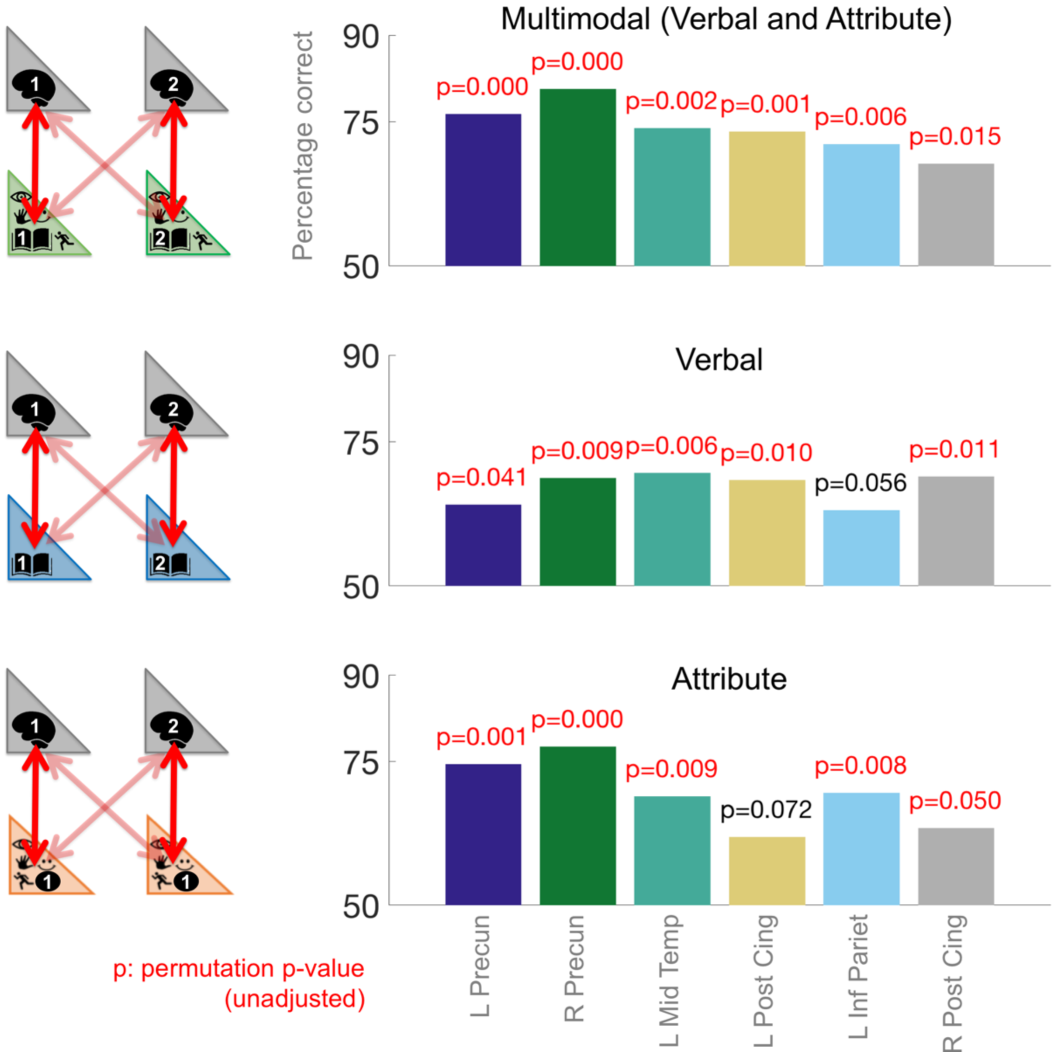

Supplementary Figure 11. Replication of Figure 6 "Individual identity can be decoded from fMRI activity elicited in imagining personal experiences" when *differently* the analysis was performed on 50 rather than 100 voxels per ROI. Tests were repeated for each pairwise combination of the 26 participants. The six ROIs tested correspond to those identified in **Supplementary Figure 6**. Each bar illustrates the percentage of times that participant-specific models better predicted the same participant's fMRI representations than another participants' fMRI data (see **Figure 2** and main text for details). P-values were estimated using permutation tests (see **Methods**) and are uncorrected. Source data are provided as a Source Data file.

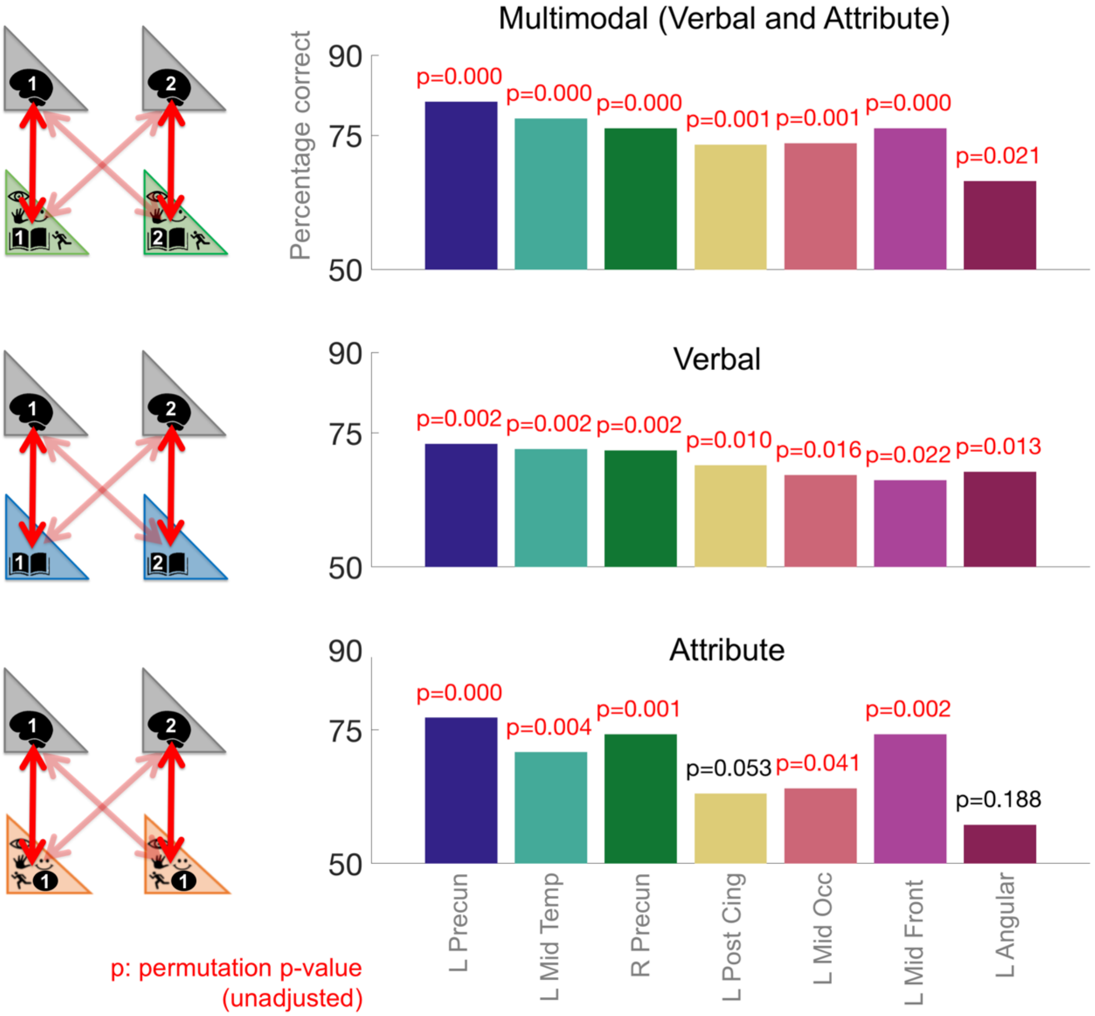

Supplementary Figure 12. Replication of Figure 6 "Individual identity can be decoded from fMRI activity elicited in imagining personal experiences" when *differently* the analysis was performed on 200 rather than 100 voxels per ROI. Tests were repeated for each pairwise combination of the 26 participants. The seven ROIs tested correspond to those identified in **Supplementary Figure 7**. Each bar illustrates the percentage of times that participant-specific models better predicted the same participant's fMRI representations than another participants' fMRI data (see **Figure 2** and main text for details). Complete results for all ROIs are in **Supplementary Table 2.** P-values were estimated using permutation tests (see **Methods**) and are uncorrected. Source data are provided as a Source Data file.

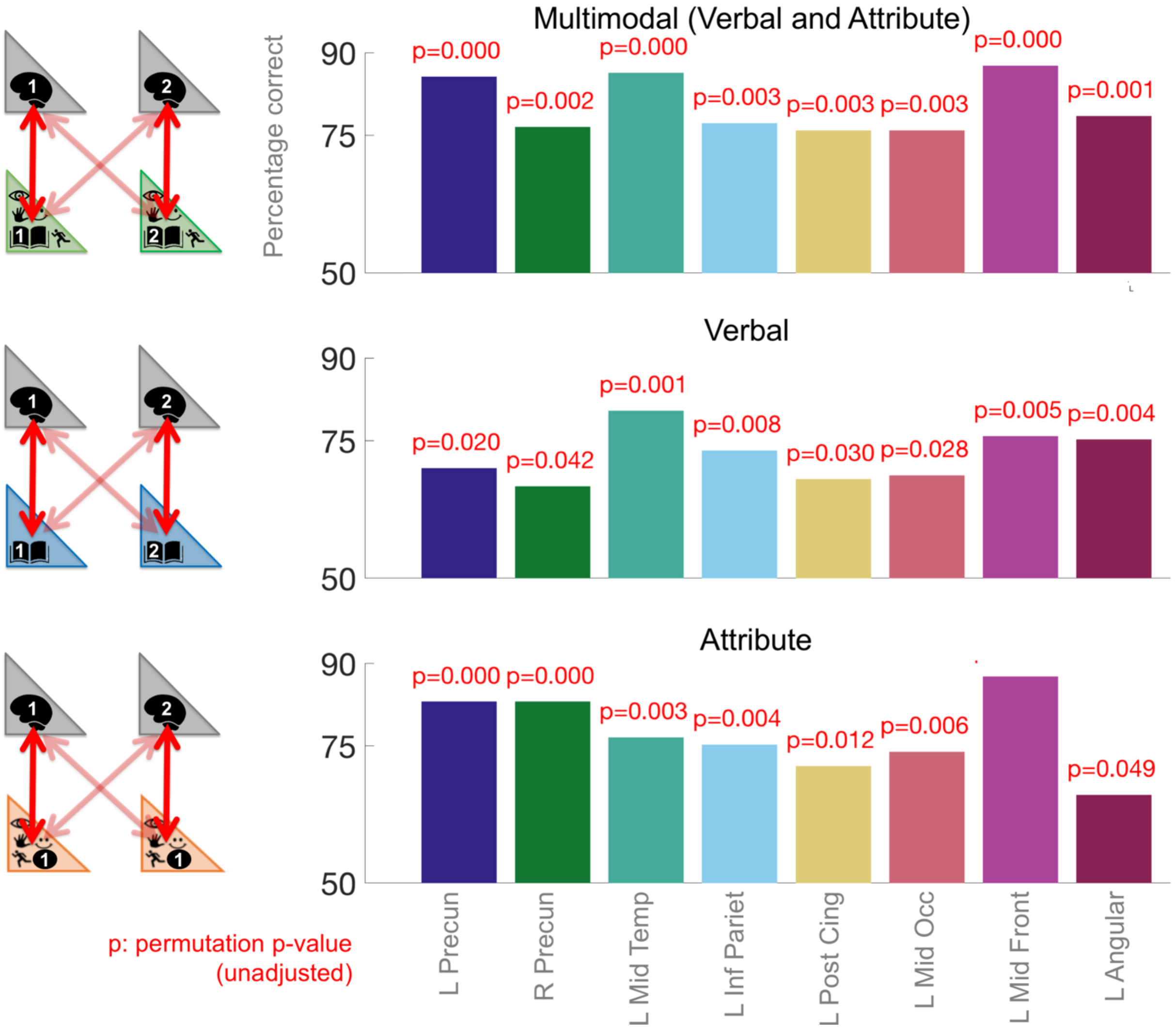

Supplementary Figure 13. Replication of Figure 6 "Individual identity can be decoded from fMRI activity elicited in imagining personal experiences" when differently the analysis was performed on the subset of 18 participants with "high fMRI signal". Participants with "high signal" were those who yielded at least one significant RSA coefficient (p<.05 uncorrected) across all 8 ROIs, when their fMRI data was compared to their personal multimodal model (see **Figure 4** caption). Each bar illustrates the percentage of times that participant-specific models better predicted the same participant's fMRI representations than another participants' fMRI data (see **Figure 2** and main text for details). The eight ROIs illustrated were identified in **Figure 4**. Complete results for all ROIs are in **Supplementary Table 2.** P-values were estimated using permutation tests (see **Methods**) and are uncorrected. Source data are provided as a Source Data file.

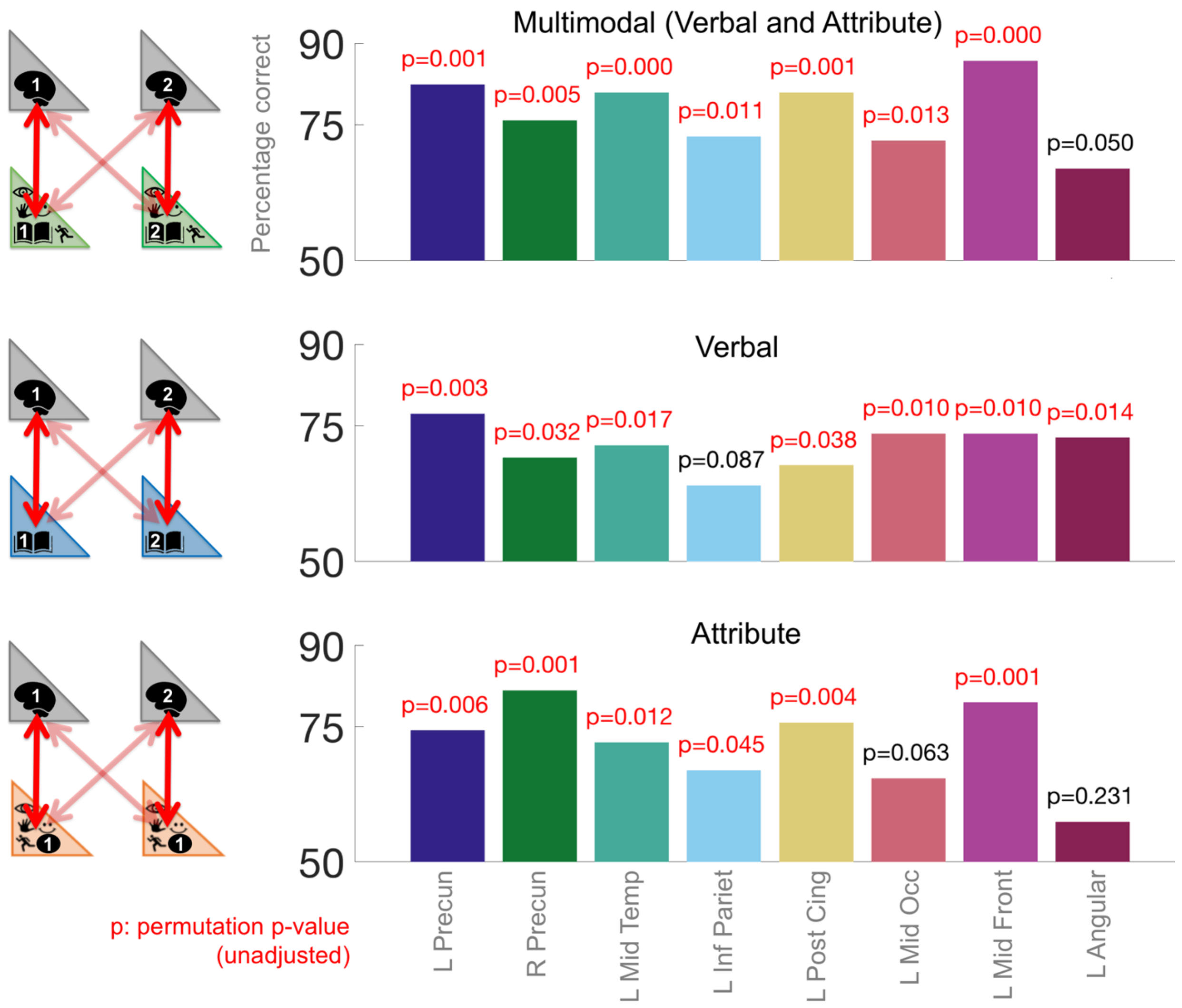

Supplementary Figure 14. Replication of Figure 6 "Individual identity can be decoded from fMRI activity elicited in imagining personal experiences" when *differently* the analysis was performed on the subset of 17 females. Each bar illustrates the percentage of times that participant-specific models better predicted the same participant's fMRI representations than another participants' fMRI data (see **Figure 2** and main text for details). The eight ROIs illustrated were identified in **Figure 4**. Complete results for all ROIs are in **Supplementary Table 2.** P-values were estimated using permutation tests (see **Methods**) and are uncorrected. Source data are provided as a Source Data file.

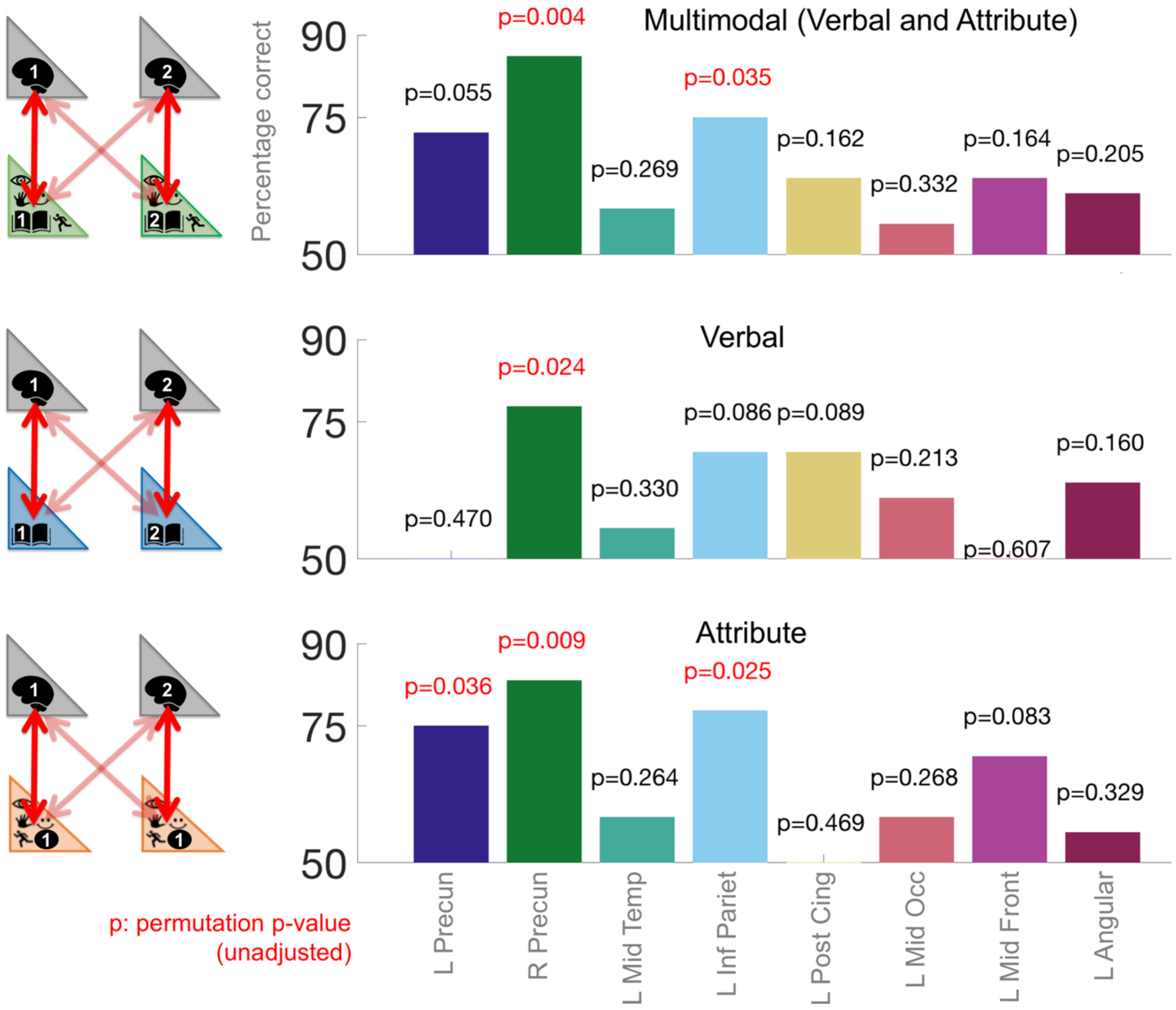

Supplementary Figure 15. Replication of Figure 6 "Individual identity can be decoded from fMRI activity elicited in imagining personal experiences" when *differently* the analysis was performed on the subset of 9 males. Results show a broadly significant trend to previous, though statistical significance estimates are weaker, reflecting the lower power associated with testing fewer participants (e.g. 9 rather than 26 in **Figure 6**). Each bar illustrates the percentage of times that participant-specific models better predicted the same participant's fMRI representations than another participants' fMRI data (see **Figure 2** and main text for details). The eight ROIs illustrated were identified in **Figure 3**. Complete results for all ROIs are in **Supplementary Table 2.** P-values were estimated using permutation tests (see **Methods**) and are uncorrected. Source data are provided as a Source Data file.

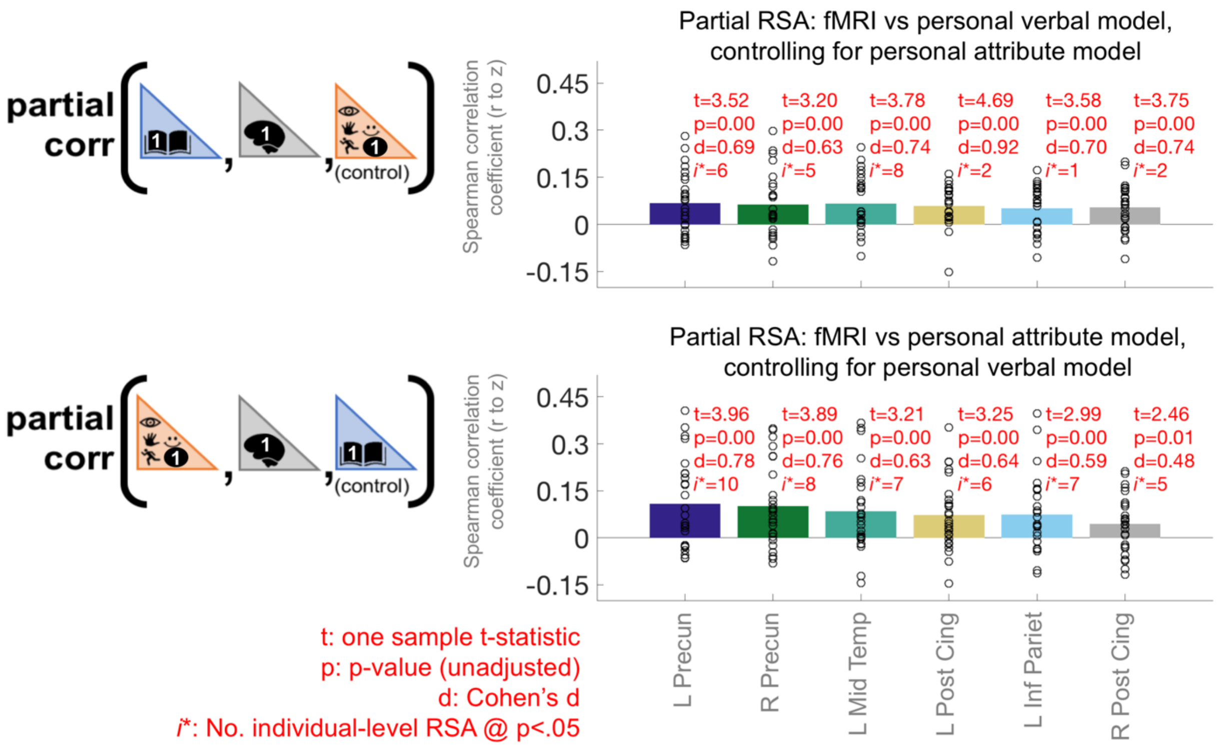

Supplementary Figure 16. Replication of Supplementary Figure 2 "Both the verbal and attribute models contributed to explaining fMRI activity in each ROI" when differently analyses were performed using 50 rather than 100 voxels per ROI. The bar plots illustrate the unique contribution that individual-level verbal or attribute models made to explaining fMRI representations in each of the six ROIs identified in **Supplementary Figure 6.** Open black circles illustrate Partial RSA coefficients for each of the 26 participants. Bar heights correspond to the mean value across participants. t-tests were one sample and against zero (1-tail). P-values are uncorrected. Exact p-values for the verbal model in the same order as plotted above were: 0.0008, 0.0019, 0.0004, 0.0000, 0.0007, 0.0005. Exact p-values for the attribute model were: 0.0003, 0.0003, 0.0018, 0.0016, 0.0031, 0.0106. Cohen's d was computed by dividing the t-statistic by $26^{1/2}$ (corresponding to 26 participants). *i** identifies the number of individual-level RSA permutation p-values < 0.05 (maximum 26, see **Methods** for details). To provide extra context for the illustrated RSA coefficients, we computed RSA between person-specific verbal and attribute models within each participant. This yielded a mean±SEM Spearman correlation coefficient of 0.23±0.02 across the 26 participants, which was significantly greater than zero (t=13.419, d=2.6, p=3e-13, one sample t-test) reflecting a strong overlap in information content across verbal/attribute models. Source data are provided as a Source Data file.

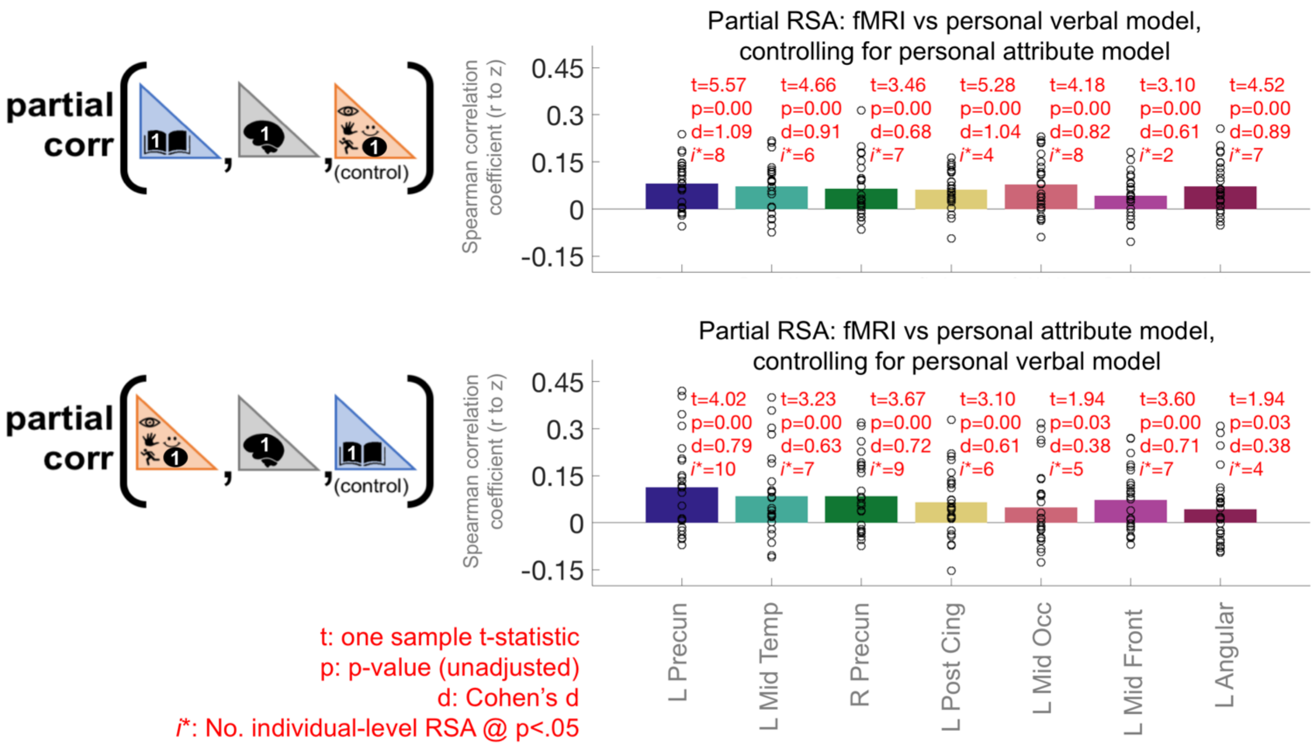

Supplementary Figure 17. Replication of Supplementary Figure 2 "Both the verbal and attribute models contributed to explaining fMRI activity in each ROI" when differently analyses were performed using 200 rather than 100 voxels per ROI. The bar plots illustrate the unique contribution that individual-level verbal or attribute models made to explaining fMRI representations in each of the seven ROIs identified in **Supplementary Figure 7.** Open black circles illustrate Partial RSA coefficients for each of the 26 participants. Bar heights correspond to the mean value across participants. t-tests were one sample and against zero (1-tail). P-values are uncorrected. Exact p-values for the verbal model in the same order as plotted above were: 0.0000, 0.0000, 0.0010, 0.0000, 0.0002, 0.0024, 0.0001. Exact p-values for the attribute model were: 0.0002, 0.0017, 0.0006, 0.0024, 0.0317, 0.0007, 0.0321. Cohen's d was computed by dividing the t-statistic by $26^{1/2}$ (corresponding to 26 participants). *i** identifies the number of individual-level RSA permutation p-values < 0.05 (maximum 26, see **Methods** for details). To provide extra context for the illustrated RSA coefficients, we computed RSA between person-specific verbal and attribute models within each participant. This yielded a mean±SEM Spearman correlation coefficient of 0.23±0.02 across the 26 participants, which was significantly greater than zero (t=13.419, d=2.6, p=3e-13, one sample t-test) reflecting a strong overlap in information content across verbal/attribute models. Source data are provided as a Source Data file.

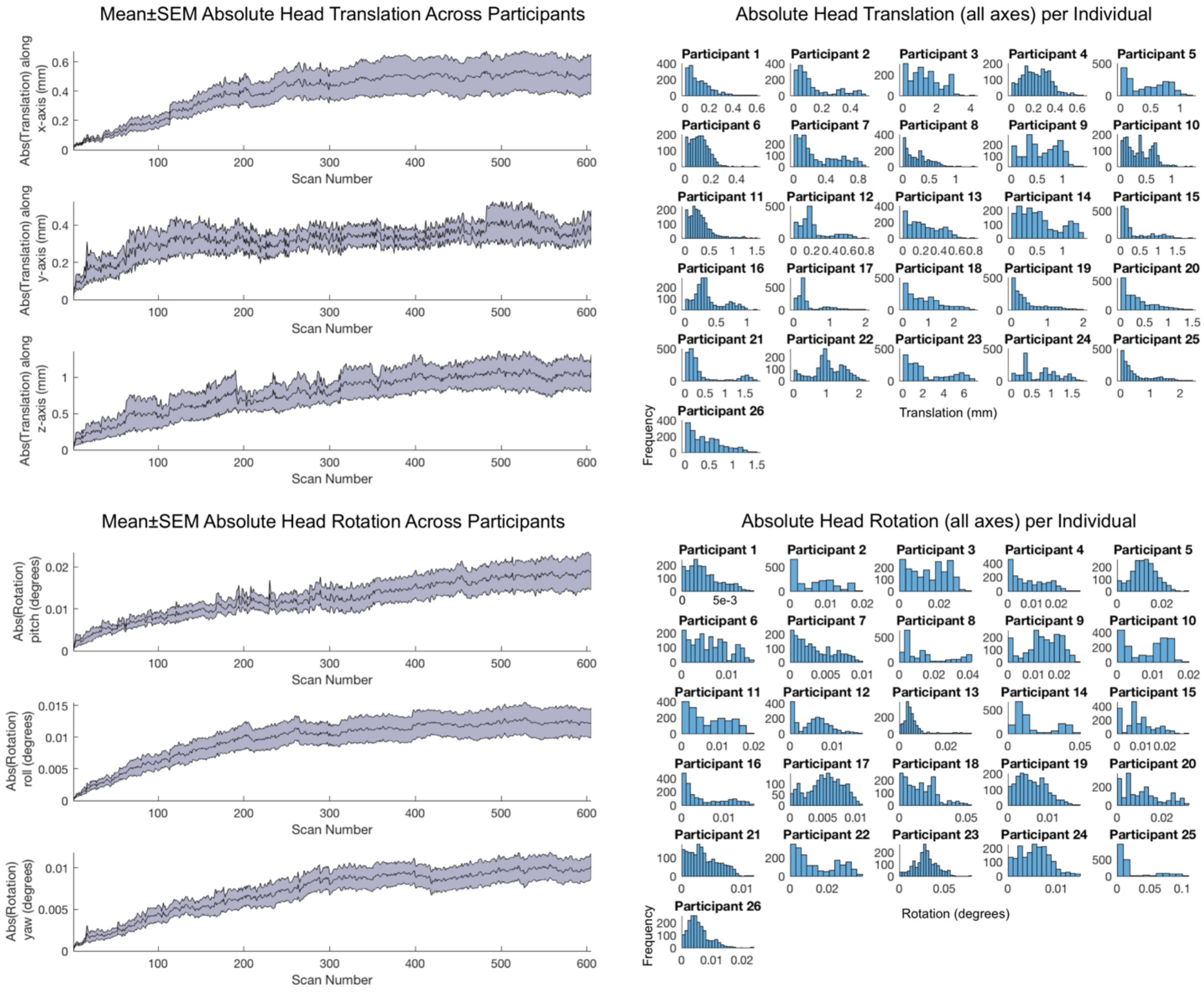

Supplementary Figure 18. Estimated head translation and rotation across the duration of the experiment.

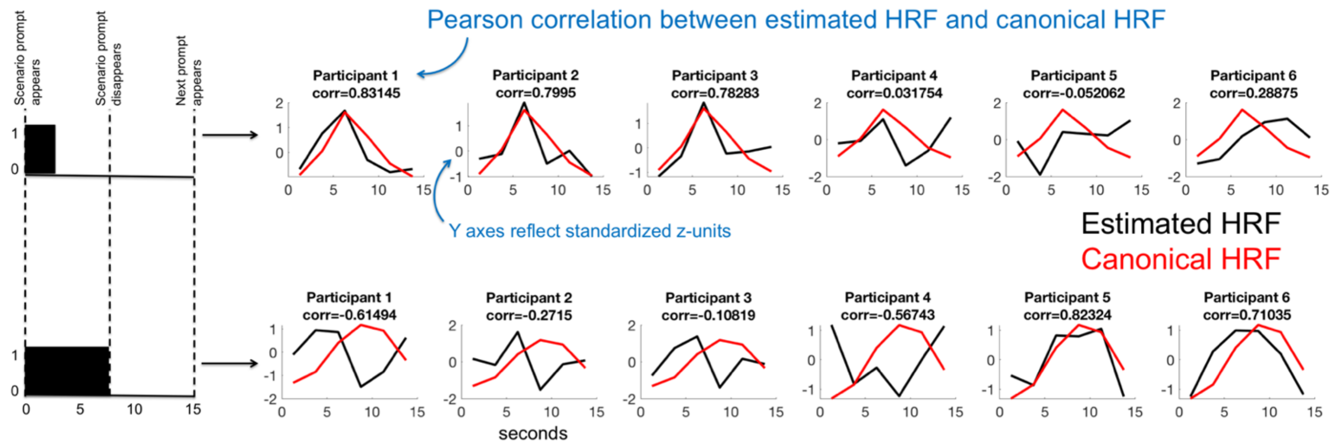

Supplementary Figure 19. Different participants were characterized by different hemodynamic response functions (HRF). We conjectured that for the current episodic simulation task HRFs would differ between people. If true this would challenge the validity of modeling the current fMRI data using the same canonical HRF for each person. To test this, we separately estimated HRFs for each individual using multiple regression to predict voxel activation based on a time-lagged stimulus representation*. The resultant beta-weights at different time lags estimate the HRF unfolding over time. We explored two ways of modeling the visual stimuli: (1) as an onset "spike" (top left), or as a "boxcar" reflecting when the stimulus was on display (bottom left). HRFs were separately estimated for each of the 20 scenarios, within each run. First, a separate stimulus timeseries was created for each scenario, within each run, at the same sample rate as fMRI (2.5sec). Ones were entered to mark stimulus display (spike/boxcar), the rest of the vector was zeros. To account for hemodynamic delays the vector was copied 6 times (reflecting the inter-stimulus interval), and each copy was temporally offset by one TR greater than the previous. Thus, if vector 1 had a one in position three, then vector 2 would have a one in position four. The vectors were concatenated into a 6column matrix. 6 head motion parameters and linear trend were concatenated with this matrix. Voxel activation time series were also represented as column vectors. Both fMRI and stimulus matrices were normalized so that each column had mean 0 and SD 1. To estimate person-specific HRFs, each voxel's activity was separately regressed on the stimulus matrix. This was repeated for each stimulus (scenario per run). To counteract overfitting, ridge regression was used (penalty=1). Beta-weights estimated the magnitude of each voxel's hemodynamic response across the 6 volumes post stimulus onset. Beta weight profiles were averaged across all scenarios and then across all voxels in Left Precuneus (which yielded strong results in our main analyses e.g. **Figure 3**). Participants' estimated HRFs are plotted in black. Overlaid in red is a canonical HRF computed by convolving a spike/boxcar with spm12 function: spm_hrf(2.5). Qualitative inspection suggests that different participants were characterized by different HRF latencies and durations. Critically, the canonical "spike" HRF weakly reflected the HRF estimated for P4 to P6. The canonical "boxcar" HRF was a poor match for P1 to P4. This provides evidence that using one canonical HRF to model all participants would result in a weak fit to many participants data. See **Supp. Figures 20/21** for complete results.

* For an illustration of a similar approach see: Broderick MP, Anderson AJ, Di Liberto GM, Crosse MJ, Lalor EC. 2018. Electrophysiological correlates of semantic dissimilarity reflect the comprehension of natural, narrative speech. Current Biology. 28(5):803-9.

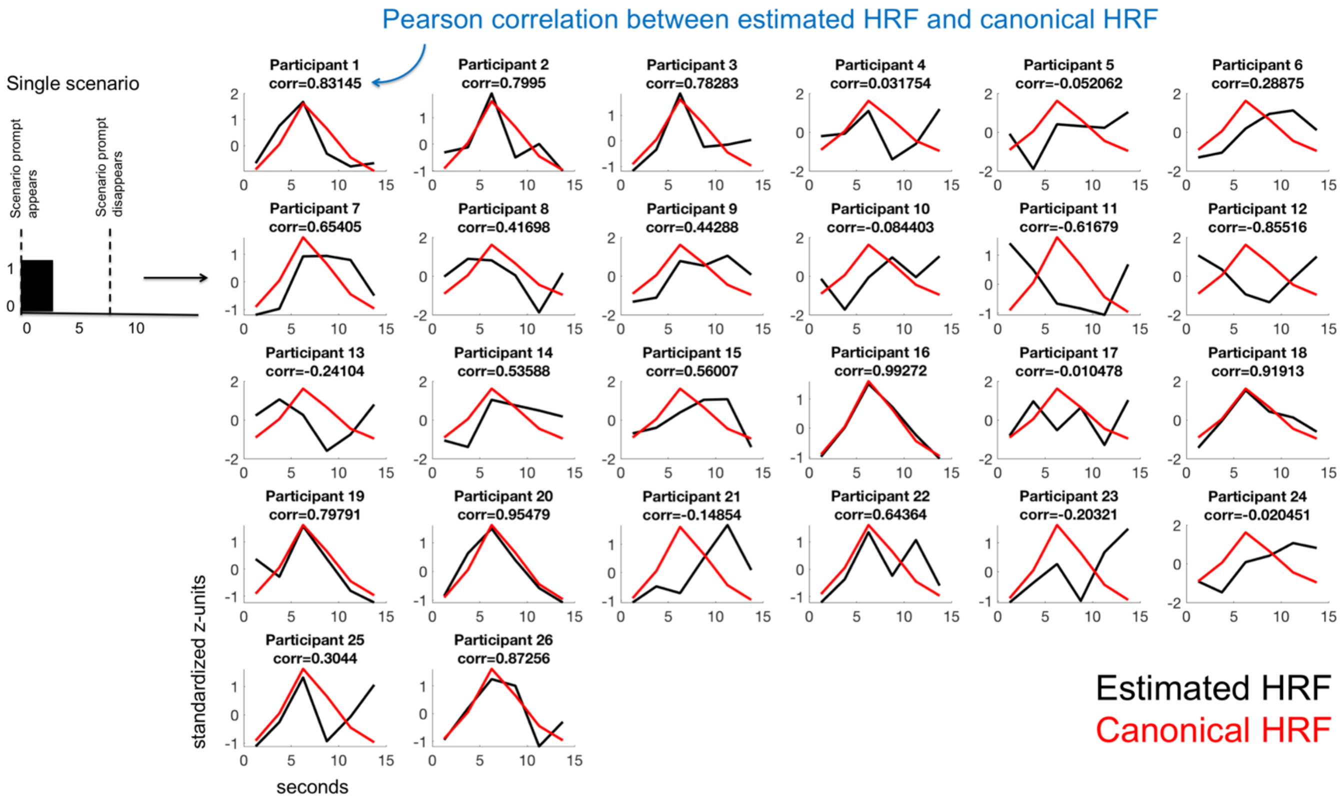

Supplementary Figure 20. Different participants were characterized by different hemodynamic response functions (HRF). The plot replicates the top row (spike) of **Supplementary Figure 19**, this time showing data for all 26 participants.

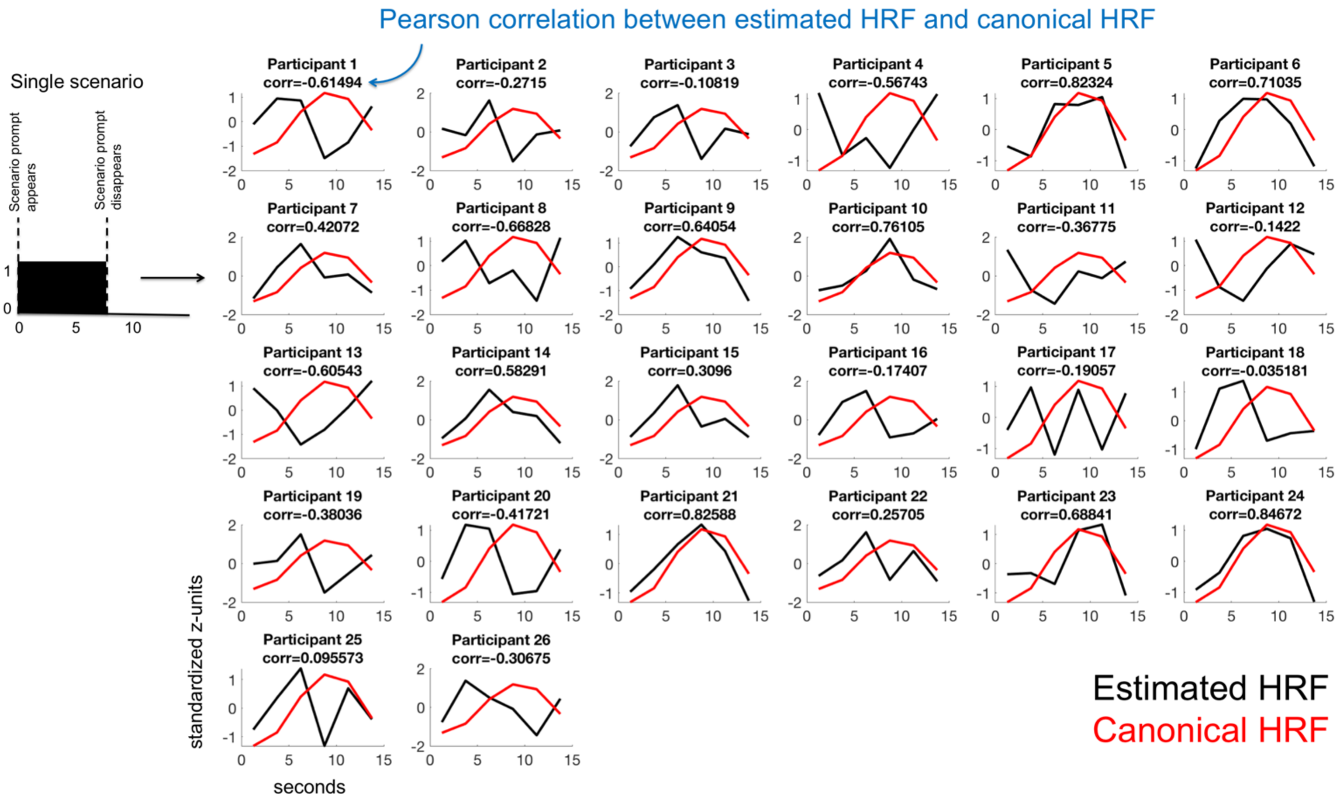

Supplementary Figure 21. Different participants were characterized by different hemodynamic response functions (HRF). The plot replicates the bottom row (boxcar) of **Supplementary Figure 19**, this time showing data for all 26 participants.

Supplementary Table 6. Detailed protocol for rating experiential attributes and scenario vividness and likelihood

Bright: Please rate the degree to which your imagined scenario contains light or brightness. Example high score: 6; The sun is very bright. Example medium score: 3; When something glows it gives off a soft light. Example low score: 0; When something gives off no light.

Color: Please rate to what degree you think of each scenario as involving color or changes in color. Example high score: 6; To blush is to turn red. Example medium score: 3; Decorating can involve adding or changing a color. Example low score: 0; Color does not play a role.

Motion: Please rate to what degree you think of each scenario as involving a specific type or a large amount of visible movement. Example high score: 6; When something bounces it is constantly moving. Example medium score: 3; Clock parts move, but not very noticeably. Example low score: 0; The scene is still.

Touch: Please rate to what degree each of your scenarios involves an action or activity in which something is felt by touch. Example high score: 6; Caressing means touching something softly. Example medium score: 3; Perceiving something may in some cases involve touching it. Example low score: 0; Touch does not play a role.

Audition: Please rate to what degree each of your scenarios involves hearing something. Example high score: 6; When something beeps it makes a sound. Example medium score: 3; Perceiving something may involve sound. Example low score: 0; The scenario involves no sound.

Music: Please rate to what degree each of your scenarios involves music or musical sounds. Example high score: 6; Singing creates musical sounds. Example medium score: 3; Chiming noises can be somewhat musical. Example low score: 0; The scenario involves no musical sounds

Speech: Please rate the degree to which each of your scenarios involves human speech sounds. Example high score: 6; Talking involves human speech sounds. Example medium score: 3; Babbling often refers to human speech that is hard to understand. Example low score: 0; The scenario involves no human speech sounds.

Taste: Please rate to what degree each of your scenarios involves tasting something. Example high score: 6; Sipping involves tasting a beverage. Example medium score: 3; Cooking is often accompanied by tasting. Example low score: 0; Taste does not play a role in this scenario.

Head: Please rate to what degree each of your scenarios involves the use of the face, mouth, or tongue. Example high score: 6; Smiling is an action involving the face and mouth. Example medium score: 3; Breathing involves the mouth or nose, although they don't actually move. Example low score: 0; The head does not play a role in this scenario.

Upper Limbs: Please rate to what degree each of your scenarios involves the use of the arms, hands, or fingers. Example high score: 6; Applauding is an action involving the arms and hands. Example medium score: 3; Jogging

usually involves the arms to some degree. Example low score: 0; The upper limbs do not play a role in this scenario.

Lower Limbs: Please rate to what degree each of your scenarios involves the use of the leg(s) or feet. Example high score: 6; Jumping requires using your legs and feet. Example medium score: 3; Sitting involves some minimal positioning of the legs. Example low score: 0; The lower limbs do not play a role in this scenarios

Path: Please rate to what degree each of your scenarios involves someone or something moving from one location to another. Example high score: 6; Traveling involves going from one place to another. Example medium score: 3; Searching may or may not require you to change your location. Example low score: 0; The scenario does not involve moving around.

Landmark: Please rate to what degree each of your scenarios involves an action or activity that occurs at a fixed location, as on a map. Example high score: 6; Libraries and other buildings have a very fixed location. Example medium score: 3; Bushes have a fixed location but are not distinctive enough to be marked on maps. Example low score: 0; The imagined scenario could happen anywhere.

Time: Please rate to what degree each of your scenarios involves an occurrence at a typical or predictable time. Example high score: 6; Waking up is something you do at a certain time of the day. Example medium score: 3; Cooking is something that often occurs in the evening. Example low score: 0; The scenario does not occur at a specific time.

Social: Please rate to what degree each of your scenarios involves interactions between people. Example high score: 6; Collaborating requires interactions between people. Example medium score: 3; Driving in a car is often done with other people. Example low score: 0; The scenario does not involve other people.

Communication: Please rate to what degree each of your scenarios involves communication or transmitting/receiving information. Example high score: 6; Explaining is when a person clarifies by communicating information. Example medium score: 3; Painting may be an artistic form of communication. Example low score: 0; The scenario does not involve communication.

Cognition: Please rate to what degree each of your scenarios involve a mental activity or state of mind that involves thinking. Example high score: 6; Considering something involves thinking about it. Example medium score: 3; Grieving is a state of mind that involves some degree of thinking. Example low score: 0; The scenario does not involve thinking.

Pleasant: Please rate to what degree each of your scenarios involves something that is pleasant. Example high score: 6; Relaxing is probably something you find pleasant. Example medium score: 3; Conversing is probably something you find somewhat pleasant. Example low score: 0; The scenario does not involve anything pleasant.

Unpleasant: Please rate to what degree each of your scenarios involves something that is unpleasant. Example high score: 6; Arguing is something you probably find unpleasant. Example medium score: 3; waiting is probably

something you find somewhat unpleasant. Example low score: 0; the scenario does not involve anything unpleasant.

Body: Please rate to what degree each of your scenarios involves visible movements of the body or limbs. Example high score: 6; Running produces visible movements of the body. Example medium score: 3; Shuddering produces small visible movements of the body.

Vividness: Please rate how vivid, or how well you are able to picture each of your scenarios. Example high score: 6; Very vivid mental image. Example medium score: 3; Some mental image. Example low score: 0; Not able to create a vivid mental image.

Likelihood: Please rate the likelihood of each scenario, or how likely it is to happen. Example high score: 6; This scenario has happened to me. Example medium score: 3; It might or could happen. Example low score: 0; This is a fictitious situation.
