# Supplementary material for: Decoding individual identity from brain activity elicited in imagining common experiences
Source: Nat Commun. 2020 Nov 20;11:5916. doi: 10.1038/s41467-020-19630-y (PMC7679397; doi:10.1038/s41467-020-19630-y)
Supplement: Supplementary file 3 — Reporting Summary [file 41467_2020_19630_MOESM3_ESM.pdf]

nature research

Corresponding author(s): andrew anderson

Last updated by author(s): July 14 2020

Reporting Summary

Nature Research wishes to improve the reproducibility of the work that we publish. This form provides structure for consistency and transparency in reporting. For further information on Nature Research policies, see our Editorial Policies and the Editorial Policy Checklist.

Statistics

For all statistical analyses, confirm that the following items are present in the figure legend, table legend, main text, or Methods section.

n/a	Confirmed	
☐	☒	The exact sample size (*n*) for each experimental group/condition, given as a discrete number and unit of measurement
☐	☒	A statement on whether measurements were taken from distinct samples or whether the same sample was measured repeatedly
☐	☒	The statistical test(s) used AND whether they are one- or two-sided<br>*Only common tests should be described solely by name; describe more complex techniques in the Methods section.*
☐	☒	A description of all covariates tested
☐	☒	A description of any assumptions or corrections, such as tests of normality and adjustment for multiple comparisons
☐	☒	A full description of the statistical parameters including central tendency (e.g. means) or other basic estimates (e.g. regression coefficient) AND variation (e.g. standard deviation) or associated estimates of uncertainty (e.g. confidence intervals)
☐	☒	For null hypothesis testing, the test statistic (e.g. *F*, *t*, *r*) with confidence intervals, effect sizes, degrees of freedom and *P* value noted<br>*Give P values as exact values whenever suitable.*
☒	☐	For Bayesian analysis, information on the choice of priors and Markov chain Monte Carlo settings
☒	☐	For hierarchical and complex designs, identification of the appropriate level for tests and full reporting of outcomes
☐	☒	Estimates of effect sizes (e.g. Cohen's *d*, Pearson's *r*), indicating how they were calculated

Our web collection on statistics for biologists contains articles on many of the points above.

Software and code

Policy information about availability of computer code

Data collection	MATLAB R2018B and Psychtoolbox were used for stimulus presentation.
Data analysis	MATLAB R2018B, spm version 12, purpose written code to recreate all analyses and regenerate is available at: https://osf.io/2e7f4/?view_only=9b474144c03d45aca62d7a71a34093f5

For manuscripts utilizing custom algorithms or software that are central to the research but not yet described in published literature, software must be made available to editors and reviewers. We strongly encourage code deposition in a community repository (e.g. GitHub). See the Nature Research guidelines for submitting code & software for further information.

Data

Policy information about availability of data

All manuscripts must include a data availability statement. This statement should provide the following information, where applicable:

- Accession codes, unique identifiers, or web links for publicly available datasets
- A list of figures that have associated raw data
- A description of any restrictions on data availability

Data to recreate all analyses are available at:
https://osf.io/2e7f4/?view_only=9b474144c03d45aca62d7a71a34093f5

Field-specific reporting

Please select the one below that is the best fit for your research. If you are not sure, read the appropriate sections before making your selection.

- [ ] Life sciences
- [x] Behavioural & social sciences
- [ ] Ecological, evolutionary & environmental sciences

For a reference copy of the document with all sections, see nature.com/documents/nr-reporting-summary-flat.pdf

Behavioural & social sciences study design

All studies must disclose on these points even when the disclosure is negative.

Study description	Data are quantitative (fMRI) and qualitative (e.g. verbal descriptions of imagination), but all qualitative measures are transformed into quantitative measures using computational methods.
Research sample	The research sample was collected at convenience from the local community or Rochester who volunteered to take part in the experiment.
Sampling strategy	A mechanistic pilot randomized controlled trial was conducted.
Data collection	MRI data was collected 3T Siemens Prisma scanner (Erlangen, Germany), using a MAC desktop for stimulus presentation, and behavioral data was collected with a laptop computer by a researcher was not blind to the study hypothesis. No-one other than researcher and participant were present for fMRI or behavioral data collection.
Timing	14th Dec 2017 to 18th Dec 2018.
Data exclusions	Of the 30 participants recruited, 26 yielded viable fMRI data: 2 participants failed to attend fMRI and 2 attended but failed to complete the experiment.
Non-participation	2 participants did not attend for unstated reasons.
Randomization	The participants were not grouped for the main analysis, which hinged around individual-level comparisons between individual's model and fMRI data.

Reporting for specific materials, systems and methods

We require information from authors about some types of materials, experimental systems and methods used in many studies. Here, indicate whether each material, system or method listed is relevant to your study. If you are not sure if a list item applies to your research, read the appropriate section before selecting a response.

Materials & experimental systems

n/a	Involved in the study
x	Antibodies
x	Eukaryotic cell lines
x	Palaeontology and archaeology
	Animals and other organisms
	x Human research participants
x	Clinical data
x	Dual use research of concern

Methods

n/a	Involved in the study
x	ChIP-seq
x	Flow cytometry
	x MRI-based neuroimaging

Animals and other organisms

Policy information about studies involving animals; ARRIVE guidelines recommended for reporting animal research

Laboratory animals	*For laboratory animals, report species, strain, sex and age OR state that the study did not involve laboratory animals.*
Wild animals	*Provide details on animals observed in or captured in the field; report species, sex and age where possible. Describe how animals were caught and transported and what happened to captive animals after the study (if killed, explain why and describe method; if released, say where and when) OR state that the study did not involve wild animals.*
Field-collected samples	*For laboratory work with field-collected samples, describe all relevant parameters such as housing, maintenance, temperature, photoperiod and end-of-experiment protocol OR state that the study did not involve samples collected from the field.*
Ethics oversight	*Identify the organization(s) that approved or provided guidance on the study protocol, OR state that no ethical approval or guidance was required and explain why not.*

Note that full information on the approval of the study protocol must also be provided in the manuscript.

Human research participants

Policy information about studies involving human research participants

Population characteristics	30 healthy older adults with normal cognition, indexed by Telephone Interview for Cognitive Status (TICS) ≥ 31, were recruited for the study. Participants had adequate visual (normal or corrected to normal vision) and auditory acuity for testing, were English-speaking, and community dwelling. Of the 30 participants, 26 yielded usable data: 2 participants failed to attend fMRI and 2 attended but failed to complete the experiment. Of the 26 participants included in analysis, 5 were left handed and 17 were female. Mean +/-SD age was 73±7.
Recruitment	Volunteers were recruited via local newspaper ads or community center flyers.
Ethics oversight	The study was approved by the research subject review board of the University of Rochester (RSRB00067540).

Note that full information on the approval of the study protocol must also be provided in the manuscript.

Magnetic resonance imaging

Experimental design

Design type	Event-related
Design specifications	Stimuli were presented on a screen in black Arial font (size 50) on a grey background that participants viewed the screen while in the scanner. fMRI was scanned during a single uninterrupted session, in which the 20 scenario stimuli were presented five times over (five runs). Scenario order was randomized within each run. Scenario stimulus prompts (e.g. "A dancing scenario") remained on screen for 7.5 seconds (3 TRs). The participants had been instructed to re-imagine themselves in the given scenario only when the stimulus prompt was on screen. There was a 7.5 second interval between scenario presentations, during which time a fixation cross was displayed. Runs were separated by a 15 second interval, in which a second by second countdown was displayed (e.g. "Starting run 2 in 13 seconds"), which was followed by 7.5 seconds of fixation cross preceding the first stimulus of the run.
Behavioral performance measures	Behavioral responses were not recorded during the scan. fMRI data was interrogated using a model built from participants' responses outside the scanner, which would reliably predict neural activation only if participants performed the task. The experimental protocol broadly adheres to Mitchell et al. 2008. Science.

Acquisition

Imaging type(s)	Functional, structural
Field strength	3T
Sequence & imaging parameters	Imaging data were collected at the Rochester Center for Brain Imaging using a 3T Siemens Prisma scanner (Erlangen, Germany) equipped with a 32-channel receive-only head coil. The fMRI scan began with a MPRAGE scan (TR/TE=1400/2344 ms, TI=702ms, Flip Angle=8°, FOV=256mm, matrix=256x256mm, 192 sagittal slices, slice thickness =1mm, voxel size 1×1×1mm3). fMRI data were collected using a gradient echoplanar imaging (EPI) sequence (TR/TE=2500ms/30ms, Flip Angle=85°, FOV=256mm, 90 axial slices, slice thickness=2mm, voxel size 2x2x2mm3, number of volumes = 639).
Area of acquisition	Whole brain

Diffusion MRI ☐ Used ☒ Not used

Preprocessing

Preprocessing software	SPM 12 was used to preprocess participants' structural and functional MRI data. Structural scans were coregistered and warped to a common template in MNI space using DARTEL. Scans were scans were slice-time corrected, motion-corrected, co-registered to their normalized structural images, and then warped to MNI space by applying the same transformation which normalized their structural image.
Normalization	Structural scans were coregistered and warped to a common template in MNI space using DARTEL. Scans were co-registered to their normalized structural images, and then warped to MNI space by applying the same transformation which normalized their structural image.
Normalization template	MNI305
Noise and artifact removal	Six head motion parameters (translation on x,y,z axes, and yaw, pitch and roll and linear trend were voxel-wise regressed out from the fMRI data within each of the 5 runs.

Volume censoring	Functional data corresponding to grey matter was segmented using default tissue segmentation parameters in SPM12.

Statistical modeling & inference

Model type and settings	Representational Similarity Analysis (RSA), using Pearson correlation to compute correlation matrices and Spearman correlation to correlate correlation matrices between model and fMRI.
Effect(s) tested	Spearman correlation coefficient arising from RSA, where RSA compared task-related fMRI activation patterns to a task-related model.

Specify type of analysis: ☐ Whole brain ☒ ROI-based ☐ Both

Anatomical location(s): Automated Anatomical Atlas and Searchlight (radius 3 voxels)

Statistic type for inference (See Eklund et al. 2016)	Representational similarity analysis within ROI.
Correction	False Discovery Rate (Benjamini, Yekutieli 2001)

Models & analysis

n/a	Involved in the study
☒	☐ Functional and/or effective connectivity
☒	☐ Graph analysis
☐	☒ Multivariate modeling or predictive analysis

Multivariate modeling and predictive analysis	Data were analysed using RSA using Pearson correlation to compute correlation matrices (separately for model and ROI-level fMRI data) and Spearman correlation to correlate correlation matrices between model and fMRI. Prior to this within each ROI voxels were selected using a strategy introduced by Mitchell et al. Science 2008. For each participant, and separately for each voxel, we took each pair of runs, and computed the inter-run Pearson correlation in activation across the 20 scenarios. This resulted in 10 correlation coefficients per voxel (derived through inter-correlating the five runs) that were r-to-z transformed. A single stability metric for each voxel was derived by taking the mean of the 10 coefficients.
